# A mechanical stimulation-related gene signature predicts prognosis, reflects immune microenvironment remodeling, and identifies *COL22A1* as a potential therapeutic target in lung adenocarcinoma

**DOI:** 10.3389/fimmu.2026.1918878

**Published:** 2026-09-16

**Authors:** Weihao Zhang, Yan Sun, Jin Li, Tongyou Sun, Lei Liu

**Affiliations:** 1 Graduate School, Chengde Medical University, Chengde, Hebei, China; 2 Beijing Garrison Command Haidian No. 24 Retired Cadres’ Sanatorium, People’s Liberation Army of China, Haidian, Beijing, China; 3 Health Examination Center, Chengde Central Hospital, Chengde, Hebei, China; 4 Department of Radiotherapy and Chemotherapy, Chengde Central Hospital, Chengde, Hebei, China; 5 Department of Cardiothoracic Surgery, Chengde Central Hospital, Chengde, Hebei, China

**Keywords:** COL22A1, lung adenocarcinoma, machine learning, mechanical stimulation, tumor immune microenvironment

## Abstract

**Background:**

Mechanical stimulation is a key biomechanical characteristic of the tumor microenvironment and is involved in tumor progression, immune microenvironment remodeling, and therapy resistance. This study aimed to develop a mechanical stimulation-related gene (MSRG)-based prognostic signature for survival prediction and to explore its association with the tumor immune microenvironment in lung adenocarcinoma (LUAD).

**Methods:**

We used a multifaceted approach to analyze MSRGs in LUAD. The AUCell algorithm was used to calculate mechanical stimulation scores at the single-cell level, while CellChat and NicheNet analyses were applied to reveal cell–cell communication and ligand-target regulatory patterns under high mechanical stimulation. Mechanical stimulation activity in TCGA-LUAD samples was quantified by ssGSEA, and WGCNA was then performed to identify key gene modules associated with this trait. An integrated machine learning framework was then used to develop a prognostic risk model. Immune infiltration, stromal and immune scores, immune checkpoint-related features, and immune evasion potential were further evaluated. Finally, the selected key gene, *COL22A1*, was validated using multiple approaches.

**Results:**

Analysis of scRNA-seq data demonstrated that mechanical stimulation (MS) scores varied considerably across different cell types. The high-MS subgroup showed enhanced intercellular communication, suggesting active tumor microenvironment remodeling. Furthermore, a prognostic model for LUAD with robust predictive performance was constructed using WGCNA and machine learning algorithms. Notably, the high-risk group showed reduced immune infiltration, higher tumor purity, elevated *CD276* expression, and increased TIDE scores, suggesting an immune-cold and immune-evasive microenvironment. *In vitro* experiments further showed that *COL22A1* enhanced the malignant behavior of LUAD cells, indicating its potential as a therapeutic target.

**Conclusion:**

We established an MSRG-based prognostic signature that not only predicts survival in LUAD but also reflects tumor immune microenvironment remodeling and potential immune escape. Our preliminary findings also suggest that *COL22A1* is involved in LUAD progression and may have potential as a therapeutic target.

## 1 Introduction

Lung adenocarcinoma (LUAD), a major histological subtype of non-small cell lung cancer (NSCLC), continues to impose a considerable global health burden (1). Although targeted therapy and immune checkpoint inhibition have improved treatment outcomes, durable clinical benefit remains limited in many patients. This unfavorable prognosis is closely linked to pronounced tumor heterogeneity and immune escape within the tumor microenvironment (2).

The tumor microenvironment (TME) contains not only cancer cells but also surrounding non-malignant cells, the extracellular matrix (ECM), and other acellular components. Within this complex environment, mechanical stimulation may result from matrix stiffening, the accumulation of solid stress, abnormal fluid shear stress, or tensile stress generated during rapid cell proliferation (3, 4). Tumor cells detect these mechanical cues through surface receptors, including integrins and mechanosensitive ion channels. Through mechanotransduction, these external cues are converted into biochemical signals that influence metabolic reprogramming, invasion, and migration (5). Sustained mechanical stimulation has also been linked to epithelial-mesenchymal transition (EMT), increased tumor cell motility, and reduced sensitivity to chemotherapy and radiotherapy (6). These effects may also intersect with broader stress-response and apoptosis-regulatory networks. For example, the *MALAT1*/miR-140-5p/Nrf2 axis has been implicated in cellular redox regulation under ischemia–reperfusion stress, whereas the GSK3β/ITCH/c-FLIP axis has been shown to counteract TRAIL-induced apoptosis in LUAD cells (7, 8). Mechanical cues may further alter the tumor immune milieu by changing the infiltration and activity of different immune cell populations (9–11). Clinical and experimental evidence also supports the prognostic relevance of the mechanical and stromal microenvironment in lung cancer. Increased collagen deposition and ECM stiffness have been associated with cancer stem-like properties and metastatic progression in LUAD, whereas a high stromal proportion has been linked to poorer survival in patients with resected NSCLC (12, 13). Although mechanical stimulation has received increasing attention in tumor biology, its role in LUAD and the related mechanisms are still not fully clarified.

Using AUCell, WGCNA, and machine learning together with multi-omics data, we identified mechanical stimulation-related genes (MSRGs) in LUAD and built a prognostic risk model. We further explored the association between the MSRG-based risk model and tumor immune microenvironment characteristics, including immune infiltration and immune evasion-related features. We then selected *COL22A1*, a key gene in the model, for further analysis. Patients with higher *COL22A1* expression showed poorer clinical outcomes, and *COL22A1* could promote malignant phenotypes in LUAD cells. Further analyses indicated that *COL22A1* may contribute to malignant behavior through activation of the FAK/Src pathway. These results provide insight into the molecular basis underlying LUAD prognosis and indicate the potential relevance of *COL22A1* in prognostic assessment and therapeutic targeting.

## 2 Materials and methods

### 2.1 Data acquisition

LUAD transcriptomic profiles and corresponding clinical information were collected from TCGA (14), whereas three external validation cohorts, GSE72094, GSE31210, and GSE50081, were downloaded from GEO (15–17). For single-cell analysis, we assembled a scRNA-seq dataset comprising 19 patients with LUAD using data from GSE189357 together with the dataset available on Code Ocean (https://codeocean.com/capsule/8321305/tree/v1) (18, 19). In addition, one LUAD spatial transcriptomics dataset (GSM9226190) was included from GSE307534. Genes related to the response to mechanical stimulation (GO:0009612) were downloaded from MSigDB.

### 2.2 Single-cell transcriptomic analysis

Seurat (v4.4.0) was used for quality control, normalization, PCA/t-SNE-based dimensionality reduction, cell clustering, and cell-type annotation (20). Cells were retained only when they met the following criteria: 200 ≤ nFeature_RNA ≤ 10,000, nCount_RNA ≥ 1,000, mitochondrial gene proportion ≤ 20%, and hemoglobin gene proportion ≤ 1%. Batch variation among datasets was corrected with Harmony (21).

### 2.3 AUCell analysis

The activity of the mechanical stimulation gene set was assessed with AUCell (22). Ranked gene expression profiles were used to calculate AUC scores. The cutoff determined by AUCell_exploreThresholds was 0.0627; cells with AUC scores ≥ 0.0627 and < 0.0627 were classified as high- and low-mechanical-stimulation cells, respectively. The distribution of AUC scores was visualized using t-SNE via the ggplot2 package.

### 2.4 Cell–cell communication analysis

CellChat (v1.6.1) was applied to infer cell–cell communication and characterize ligand-receptor interaction networks (23). Only interactions with permutation-based P values < 0.05 were retained, and communications involving cell populations containing fewer than 10 cells were excluded. NicheNet (v1.0.0) was subsequently used to infer signaling from high-MS fibroblasts to epithelial cells (24). Genes detected in at least 5% of sender or receiver cells were considered expressed. Candidate ligands were ranked by corrected AUPR, and the top 30 ligands were retained.

### 2.5 ssGSEA and WGCNA

Mechanical stimulation activity in each TCGA-LUAD sample was quantified by ssGSEA using the GSVA R package (25). The ssGSEA-derived mechanical stimulation score was used as a continuous trait in WGCNA. The DEGs identified between the AUCell-defined high- and low-mechanical-stimulation cells in the single-cell dataset were used as input for WGCNA. A scale-free co-expression network was subsequently constructed, and co-expression modules were identified based on topological overlap patterns and dynamic tree cutting (26). Pearson correlation analysis was performed between module eigengenes and mechanical stimulation scores. Modules with an absolute Pearson correlation coefficient greater than 0.70 and a corresponding P value less than 0.05 were defined as mechanical stimulation-associated modules and retained for subsequent analyses.

### 2.6 Construction of the prognostic signature

The overlap between DEGs in the TCGA-LUAD cohort and WGCNA-derived module genes yielded 169 candidate genes. These genes were then entered into a machine-learning pipeline to generate prognostic signatures in the TCGA-LUAD cohort. Model performance was evaluated in three external validation cohorts (GSE72094, GSE31210, and GSE50081) using Harrell’s concordance index (C-index). Among the tested algorithms, LASSO–RSF showed the strongest overall C-index performance and was therefore used for subsequent model construction. Ten-fold cross-validation was performed for LASSO feature selection using a fixed random seed of 123. The same seed was used for random survival forest training with 1,000 trees and a minimum terminal node size of 4. Model performance was assessed using Kaplan–Meier survival curves and ROC analysis (27). A nomogram for predicting 1-, 3-, and 5-year overall survival was constructed using the rms package, and calibration curves were used to evaluate its predictive consistency.

### 2.7 Analysis of tumor microenvironment immune characteristics

Immune cell infiltration in the TCGA-LUAD bulk RNA-seq cohort was estimated using four transcriptome-based deconvolution algorithms: MCP-counter, EPIC, CIBERSORT, and TIMER. Spearman correlation analysis was performed to evaluate the associations between the risk score and model gene expression and the inferred abundance of immune cell populations. ESTIMATE was used to calculate tumor purity, stromal score, immune score, and ESTIMATE score. The expression levels of selected immune checkpoint genes were compared between the low- and high-risk groups. TIDE was further applied to assess the potential for tumor immune dysfunction, exclusion, and immune evasion (28, 29).

### 2.8 Spatial transcriptomics analysis

Spatial transcriptomics data were imported using the Load10X_Spatial function and normalized using the SCTransform algorithm. Multimodal Intersection Analysis (MIA) was applied to assess the statistical enrichment of scRNA-seq-defined cell type signatures within spatial transcriptomics clusters. A hypergeometric test was used to evaluate the significance of gene set overlap and identify spatial regions associated with specific cell populations.

### 2.9 Cell line and culture conditions

BEAS-2B cells, as well as LUAD cell lines PC9 and H1975, were sourced from Servicebio (Wuhan, China). Cells were maintained in RPMI-1640 medium (Beyotime, C2721) supplemented with 10% fetal bovine serum (Beyotime, C0226S) and 1% penicillin-streptomycin (Beyotime, C0222). Cells were cultured at 37 °C in a humidified incubator with 5% CO_2_. Cell identity was authenticated by short tandem repeat (STR) profiling, and no mycoplasma contamination was detected.

### 2.10 Transfection for *COL22A1* knockdown and overexpression

*COL22A1*-specific siRNAs (si-*COL22A1*) were obtained from MedChemExpress (MCE) for transient silencing. Lipofectamine 2000 (Invitrogen, Carlsbad, CA, USA) was used for cell transfection as instructed by the manufacturer. The sequences of the *COL22A1*-targeting siRNAs are included in the **Supplementary Materials**. The plasmid for *COL22A1* overexpression was constructed by General Biosystems (Anhui) Co., Ltd. For rescue experiments, *COL22A1*-overexpressing cells were treated with Defactinib (MCE, HY-12289) for pathway inhibition before Western blot and Transwell analyses.

### 2.11 Immunofluorescence staining

Cells grown on coverslips were fixed with 4% paraformaldehyde, permeabilized using 0.1% Triton X-100, and blocked in 5% BSA. Cells were incubated with primary antibodies overnight at 4 °C, followed by incubation with fluorescent secondary antibodies. Nuclei were counterstained with DAPI (1 μg/mL) for 5 min. Fluorescence images were acquired under a fluorescence microscope.

### 2.12 Proliferation assays

Cell proliferation was evaluated by CCK-8 and EdU assays. For CCK-8 analysis, 1,000 cells were seeded into each well of 96-well plates. At 0, 24, 48, and 72 h, 10 μL of CCK-8 reagent (Beyotime, C0037) was added to each well, and OD450 values were measured after 2 h of incubation. For EdU staining, EdU working solution was added to cells cultured in 24-well plates after the indicated treatments. Following fixation and permeabilization, EdU staining was conducted with the BeyoClick™ EdU Cell Proliferation Kit (Beyotime, C0078S). DAPI was used for nuclear staining, and cell proliferation was assessed according to the fraction of cells incorporating EdU. For the colony formation assay, 1,000 cells per well were seeded into 6-well plates and cultured for 14 days under standard conditions. The colonies were then fixed with 4% paraformaldehyde for 15 min, followed by crystal violet staining for visualization and counting.

### 2.13 Transwell assays for migration and invasion

Each upper chamber received 2 × 10^4^ cells suspended in 200 μL serum-free medium, whereas the lower compartment contained 600 μL complete medium supplemented with 20% FBS. The chambers were incubated for 24–48 h before further processing. Invasion assays were performed using Matrigel-precoated chambers, with the remaining steps identical to those of the migration assay. At the end of incubation, the membranes were fixed and stained with crystal violet. Cells were counted in three randomly selected fields under an inverted microscope.

### 2.14 Cell apoptosis assay

Following the indicated treatments, both floating cells in the culture medium and adherent cells were collected. The cells were detached with 0.25% trypsin/EDTA at 37 °C for 5 min and collected after neutralization. The resulting cell suspension was washed twice with cold PBS, resuspended in binding buffer, and stained with FITC Annexin V and PI for 25 min in the dark. Apoptosis was measured using a NovoCyte 2000R flow cytometer, and the resulting data were subsequently analyzed.

### 2.15 Western blot analysis

Cells were harvested and centrifuged to obtain cell pellets. Cell pellets were lysed in RIPA buffer (Beyotime, P0013B), and total protein concentration was measured with a BCA assay kit (Beyotime, P0398S). The primary antibodies included Anti-COL22A1 (Thermo, PA5-70816), Anti-β-Tubulin (Proteintech, 66240-1-Ig), Anti-Phospho-FAK (MCE, HY-P80460), Anti-FAK (MCE, HY-P80125), Anti-Phospho-Src (MCE, HY-P86105), and Anti-Src (MCE, HY-P80338). The membranes were subsequently exposed to HRP-conjugated goat anti-rabbit or goat anti-mouse IgG antibodies (Proteintech, RGAR001/RGAM001). Immunoreactive bands were visualized with an ECL reagent (Servicebio, G2074-500ML).

### 2.16 Statistical methods

Bioinformatic analyses were performed in R software (version 4.4.3), whereas experimental data were processed and visualized using GraphPad Prism (version 10.1.2). All experiments were conducted with at least three biological replicates. Comparisons between two groups were performed using Student’s t-test or the Wilcoxon rank-sum test. Comparisons among groups were performed using one-way or two-way ANOVA, as appropriate. Survival differences were assessed by Kaplan–Meier analysis with the log-rank test. Associations between variables were examined by Pearson correlation analysis. A *p* value < 0.05 was considered statistically significant (*p < 0.05, **p < 0.01, ***p < 0.001).

## 3 Results

### 3.1 Cell type identification and mechanical stimulation score analysis

After quality-control filtering, 168,176 cells were retained for downstream analysis. scRNA-seq identified 32 initial clusters (**Figure 1A**), which were then annotated into 11 major lineages (**Figure 1B**). The heatmap displays canonical marker expression across annotated cell lineages (**Figure 1C**). Overlap analysis between mechanical stimulation-related genes (MSRGs) and differentially expressed genes (DEGs) from individual cell subpopulations identified 35 core MSRGs (**Supplementary Figure 1A**). AUCell scoring demonstrated heterogeneity in mechanical stimulation scores across different cell populations (**Figure 1D**), with epithelial cells, fibroblasts, and endothelial cells showing relatively higher scores (**Supplementary Figure 1B**). This heterogeneous distribution suggested that mechanical stimulation-related activity varied across LUAD cellular compartments.

**Figure 1:**
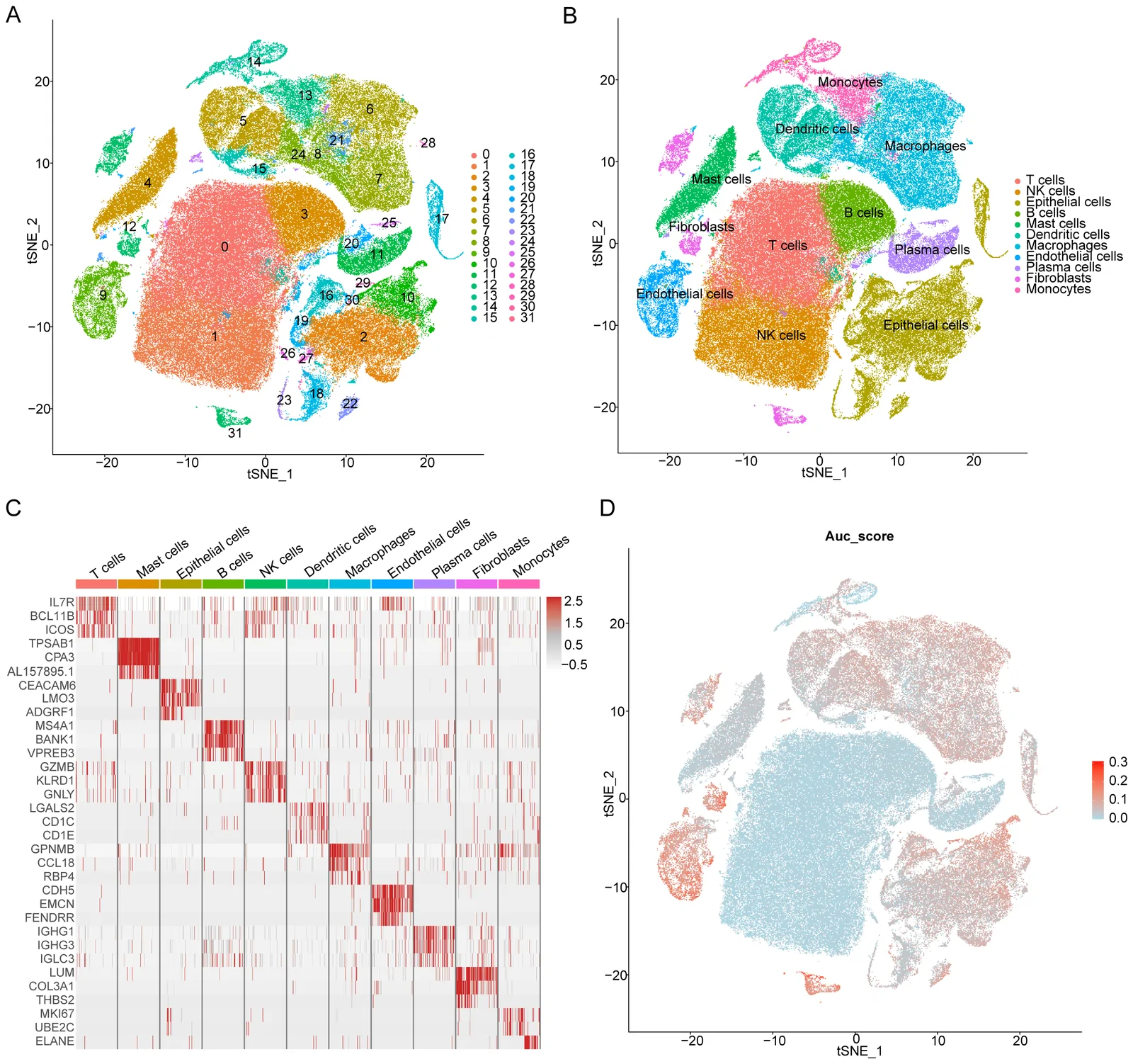
Single-cell RNA-seq landscape of LUAD. **(A)** t-SNE plot showing 32 distinct cell clusters. **(B)** Annotation of 11 major cell lineages on the t-SNE map. **(C)** Heatmap showing the top three marker genes for each cell lineage. **(D)** AUCell-based distribution of mechanical stimulation scores in the scRNA-seq dataset.

### 3.2 Cell–cell communication across MS subgroups

Given the cell-type heterogeneity in MS activity, we next asked whether the high-MS state was accompanied by coordinated changes in cell–cell communication within the tumor microenvironment. Cells were stratified into high- and low-MS groups based on mechanical stimulation scores, and their distribution on the t-SNE plot is shown in **Figure 2A**. CellChat analysis showed that the high-MS subgroup exhibited more frequent intercellular interactions and greater overall signaling activity than the low-MS subgroup (**Figure 2B**), together with a more active communication network (**Figure 2C**). Notably, thicker interaction edges were observed between fibroblasts and epithelial cells. Further analysis of outgoing and incoming signaling strengths showed that fibroblasts from the high-MS group had markedly increased outgoing signal strength, identifying them as the primary signaling source within this group (**Figure 2D**). At the signaling pathway level, information flow was enhanced across multiple pathways in the high-MS group, with pathways related to extracellular matrix (ECM) remodeling showing the most significant enrichment (**Figure 2E**).

**Figure 2:**
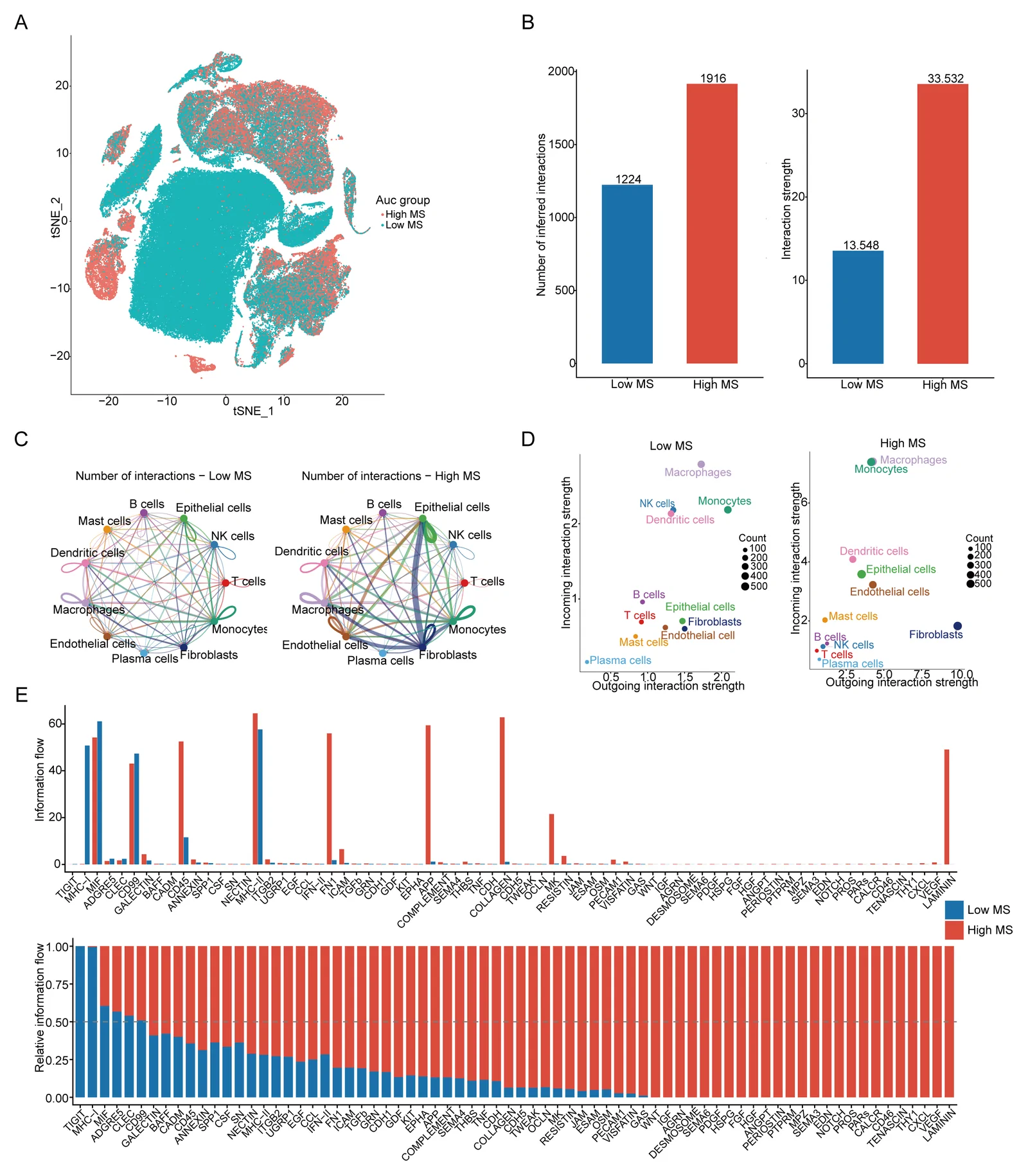
Cell–cell communication differences between high- and low-MS groups. **(A)** t-SNE map showing cell distribution in the two MS groups. **(B)** Comparison of interaction number and overall interaction strength between the low-MS and high-MS groups. **(C)** Cell–cell communication networks in the two groups. **(D)** Outgoing and incoming signaling strength of each cell type. **(E)** Pathway-level comparison of information flow, highlighting signaling pathways altered in the high-MS group.

### 3.3 NicheNet analysis

Because fibroblasts emerged as a major signaling source in the high-MS subgroup, we next investigated the potential regulatory signals transmitted from fibroblasts to epithelial cells. To further elucidate the regulatory mechanisms by which fibroblasts modulate epithelial cells under high mechanical stimulation (MS), NicheNet analysis was conducted. The results revealed that *TGFB1* exhibited the highest predicted ligand activity among fibroblast-derived candidate ligands (**Supplementary Figure 2A**). Furthermore, a ligand-target regulatory heatmap was constructed to visualize the potential downstream regulatory patterns (**Supplementary Figure 2B**). KEGG analysis of the predicted target genes indicated prominent enrichment in integrin signaling, ECM-receptor interaction, focal adhesion, and PI3K/Akt signaling (**Supplementary Figure 2C**). Correspondingly, GO analysis further linked these genes to biological processes such as wound healing and chemotaxis, cellular components including the collagen-containing extracellular matrix, and molecular functions involving ECM binding (**Supplementary Figure 2D**).

### 3.4 WGCNA-based network analysis

To further explore mechanical stimulation-related transcriptional features in bulk LUAD tumors, we examined mechanical stimulation-associated gene expression patterns in the TCGA-LUAD cohort. ssGSEA was first applied to quantify mechanical stimulation activity in TCGA-LUAD samples. Subsequently, gene modules associated with mechanical stimulation were identified by WGCNA. Scale-free topology analysis supported β = 5 as the soft-thresholding power for subsequent WGCNA network construction (**Figure 3A**). Hierarchical clustering based on gene expression similarity produced a dendrogram in which distinct colors denoted independent modules (**Figure 3B**). Module–trait correlation analysis showed the associations between each module eigengene and the mechanical stimulation score (**Figure 3C**). Based on the module-selection criteria of |r| > 0.70 and p < 0.05, the yellow module (MEyellow, *r* = 0.73, *p* = 1 × 10^-77^) and the blue module (MEblue, *r* = 0.71, *p* = 2 × 10^-73^) were identified as mechanical stimulation-associated modules, and their genes were retained for subsequent analysis. Scatter plots detailed the correlation between genes within these two modules and mechanical stimulation traits (**Figures 3D, E**). The TCGA-LUAD expression matrix was further subjected to differential expression analysis (**Figure 3F**). Overlap analysis between the DEGs and genes from the selected WGCNA modules yielded 169 candidate genes (**Figure 3G**).

**Figure 3:**
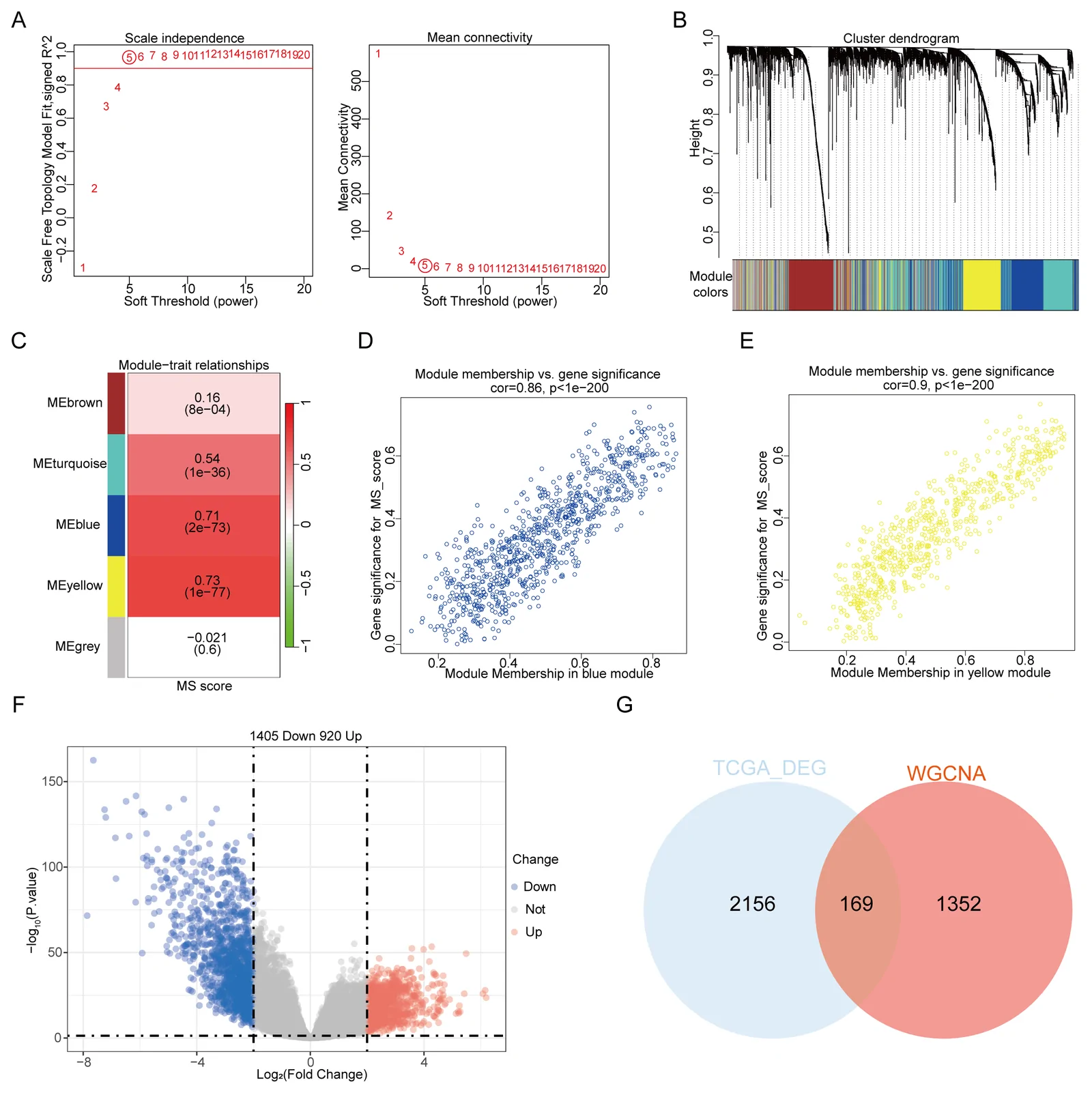
WGCNA analysis of genes associated with mechanical stimulation. **(A)** Soft-threshold selection for network construction. **(B)** Cluster dendrogram and module colors. **(C)** Relationships between modules and mechanical stimulation scores. **(D, E)** Correlation of gene significance with module membership in the blue and yellow modules. **(F)** Volcano plot showing differentially expressed genes in TCGA-LUAD. **(G)** Overlap between TCGA-LUAD DEGs and genes from the selected WGCNA modules.

### 3.5 Prognostic model construction

Univariate Cox regression of the 169 candidate genes yielded 49 prognosis-related genes for subsequent machine learning model construction. The combination of LASSO and Random Survival Forest (RSF) outperformed all other tested models, reaching the highest average C-index of 0.717 (**Figure 4A**). The LASSO-RSF model first employed LASSO regression for feature selection, with the optimal penalty parameter λ determined by ten-fold cross-validation (**Figures 4B**; **Supplementary Figure 3A**), yielding 20 candidate genes. These genes were then used to construct the RSF prognostic model, and variable importance analysis was subsequently performed (**Figures 4C**; **Supplementary Figure 3B**).

**Figure 4:**
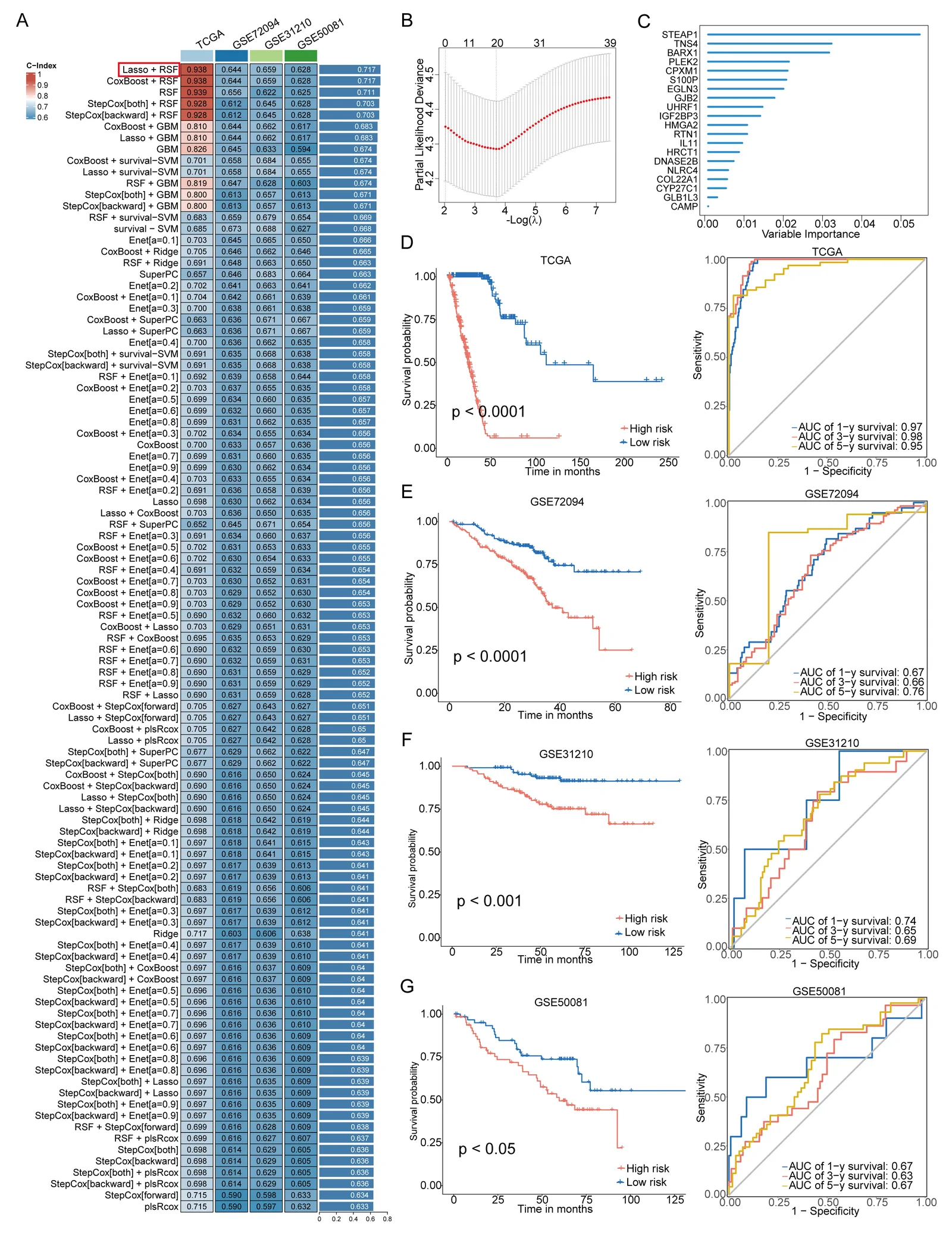
Performance of the machine learning-based prognostic model. **(A)** Comparison of 101 machine learning algorithm combinations across all cohorts using the C-index. **(B)** LASSO-based selection of survival-related genes. **(C)** Variable importance ranking derived from the RSF model. **(D–G)** Kaplan–Meier survival curves and ROC-based predictive performance across TCGA-LUAD and three external cohorts.

Each patient was assigned a risk score, and the cohort was then divided into high- and low-risk groups according to the median value. To evaluate the model’s predictive performance and robustness, Kaplan–Meier survival and receiver operating characteristic (ROC) analyses were performed in the training set (TCGA-LUAD) and three independent external validation cohorts (GSE72094, GSE31210, and GSE50081) (**Figures 4D–G**). In all cohorts, the low-risk group showed a clear survival advantage over the high-risk group in Kaplan–Meier analysis (*p* < 0.05). AUC values consistently exceeded 0.6, indicating robust model performance and potential clinical utility.

### 3.6 Nomogram performance and validation

To facilitate clinical application, we subsequently developed a nomogram incorporating the risk score and clinicopathological variables. Cox regression analyses identified pathological stage and risk score as independent predictors of overall survival in LUAD (**Supplementary Figures 4A, B**). The nomogram based on these variables showed good calibration across the three assessed time points (**Supplementary Figure 4C, D**). Higher nomogram-derived risk scores were associated with less favorable overall survival (*p* < 0.001; **Supplementary Figure 4E**). Moreover, the model achieved strong predictive performance, with all AUCs exceeding 0.90 (**Supplementary Figure 4F**).

### 3.7 Immune features of the tumor microenvironment

We next explored the immune characteristics associated with the MSRG-defined risk state. Immune infiltration profiling indicated that elevated risk scores coincided with reduced abundance of most antitumor immune cell subsets, particularly T cells and B cells (**Figure 5A**). According to the ESTIMATE results, tumors in the high-risk subgroup were characterized by higher purity and lower immune scores, consistent with an immune-cold phenotype (**Figures 5B–E**). The high-risk subgroup exhibited significantly higher *CD276* expression and increased TIDE scores compared with the low-risk subgroup (**Figures 5F, G**), suggesting a more pronounced immune-evasive phenotype.

**Figure 5:**
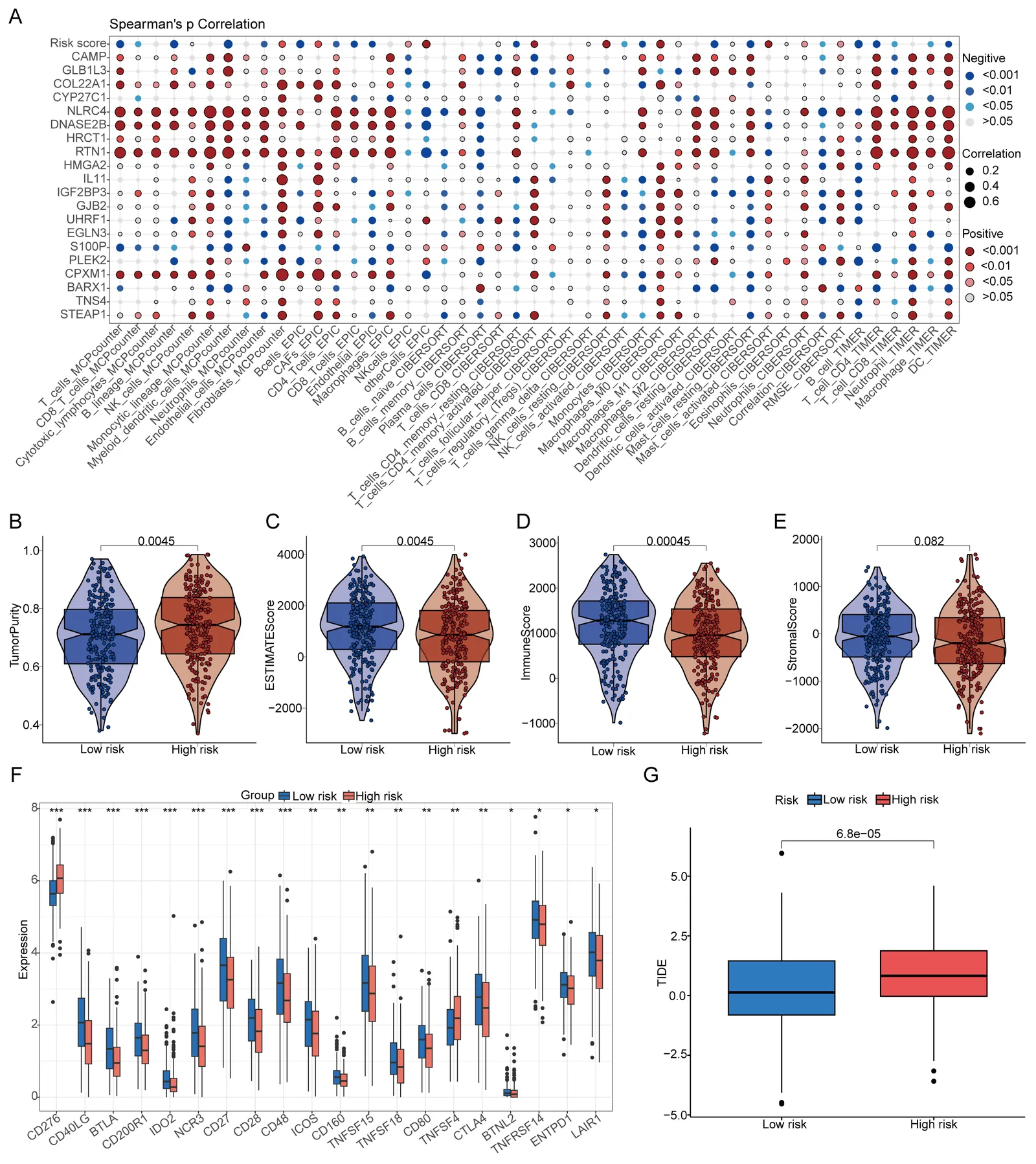
Immune microenvironment features in different risk groups. **(A)** Correlations of infiltrating immune cells with model genes and risk score based on TIMER, EPIC, MCP-counter, and CIBERSORT. **(B–E)** Tumor purity, ESTIMATE score, immune score, and stromal score in the low- and high-risk groups. **(F)** Differential expression of immune checkpoint genes between the two risk groups in TCGA-LUAD. **(G)** TIDE scores in the low- and high-risk groups. *p < 0.05; **p < 0.01; ***p < 0.001.

### 3.8 Multidimensional validation of *COL22A1*

Univariate Cox regression identified *COL22A1* as one of the genes most strongly associated with patient outcome (p < 0.001), with its hazard ratio (HR) ranking among the highest (**Figure 6A**). *COL22A1* encodes collagen XXII, an extracellular matrix component associated with mechanical stimulation responses. In LUAD, *COL22A1* expression was higher in tumor samples than in normal samples (**Figure 6B**). Clinical correlation analysis indicated that *COL22A1* levels increased with clinical stage, with significantly higher expression in Stage III–IV patients than in Stage I–II patients (*p* = 0.021; **Figure 6C**). Kaplan–Meier survival curves indicated less favorable overall survival in the *COL22A1*-high subgroup than in the *COL22A1*-low subgroup (*p* = 0.017; **Figure 6D**). Previous studies have reported aberrant *COL22A1* expression in several malignancies and suggested its association with tumor progression and poor clinical outcomes. However, the role of *COL22A1* in LUAD and the pathways through which it acts remain unclear. Therefore, *COL22A1* was selected for further experimental investigation.

**Figure 6:**
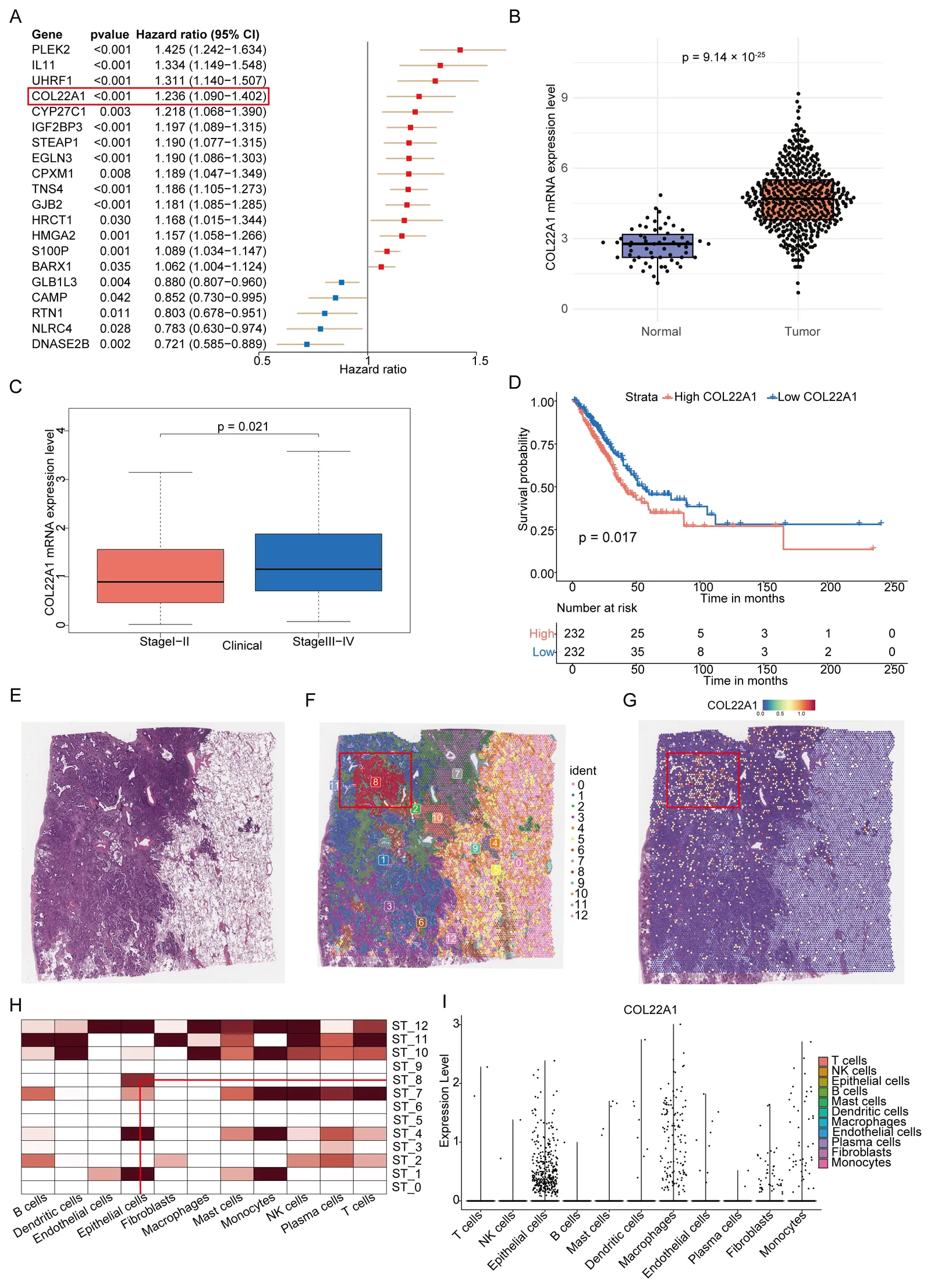
Multidimensional validation and spatial analysis of *COL22A1* in LUAD. **(A)** Univariate Cox forest plot for model candidate genes. **(B)** *COL22A1* expression in normal versus tumor tissues from TCGA-LUAD. **(C)** Box plot of *COL22A1* expression across LUAD clinical stages. **(D)** Overall survival analysis according to *COL22A1* expression level. **(E)** H&E staining showing the morphology of the spatial transcriptomics (ST) section. **(F)** Unsupervised clustering of the ST section into 13 spatial clusters (Ident 0–12). **(G)** Spatial distribution of *COL22A1* expression across the ST section. **(H)** Multimodal Intersection Analysis (MIA) heatmap. **(I)** scRNA-seq data showing *COL22A1* expression across different cell types in LUAD.

To further clarify the spatial distribution characteristics of *COL22A1*, we analyzed the LUAD spatial transcriptomics (ST) sample GSM9226190 (**Figure 6E**). Multimodal Intersection Analysis (MIA) was employed to assess the enrichment of single-cell subpopulations across the spatial transcriptomics section (**Figures 6F–H**). The results showed that the ST_8 cluster corresponded to an epithelial cell-enriched region, where *COL22A1* expression was mainly concentrated. Single-cell RNA-seq further detected *COL22A1* transcripts in epithelial cells, macrophages, fibroblasts, and monocytes (**Figure 6I**).

### 3.9 *COL22A1* promotes malignant phenotypes in LUAD cells

We next investigated the functional role of *COL22A1* in LUAD cells. We first examined COL22A1 expression and transfection efficiency in PC9 and H1975 cells. Western blot results showed that COL22A1 expression was higher in PC9 and H1975 cells than in the normal bronchial epithelial cell line BEAS-2B (**Figure 7A**). *COL22A1* was then knocked down using siRNA, and a *COL22A1* overexpression model was established. Western blot analysis showed that si-*COL22A1–*1 markedly reduced COL22A1 protein expression, whereas COL22A1 overexpression increased COL22A1 protein levels (**Figures 7B, C**). Immunofluorescence staining further showed that COL22A1 fluorescence signals were reduced after *COL22A1* knockdown and enhanced after *COL22A1* overexpression (**Figures 7D, E**).

**Figure 7:**
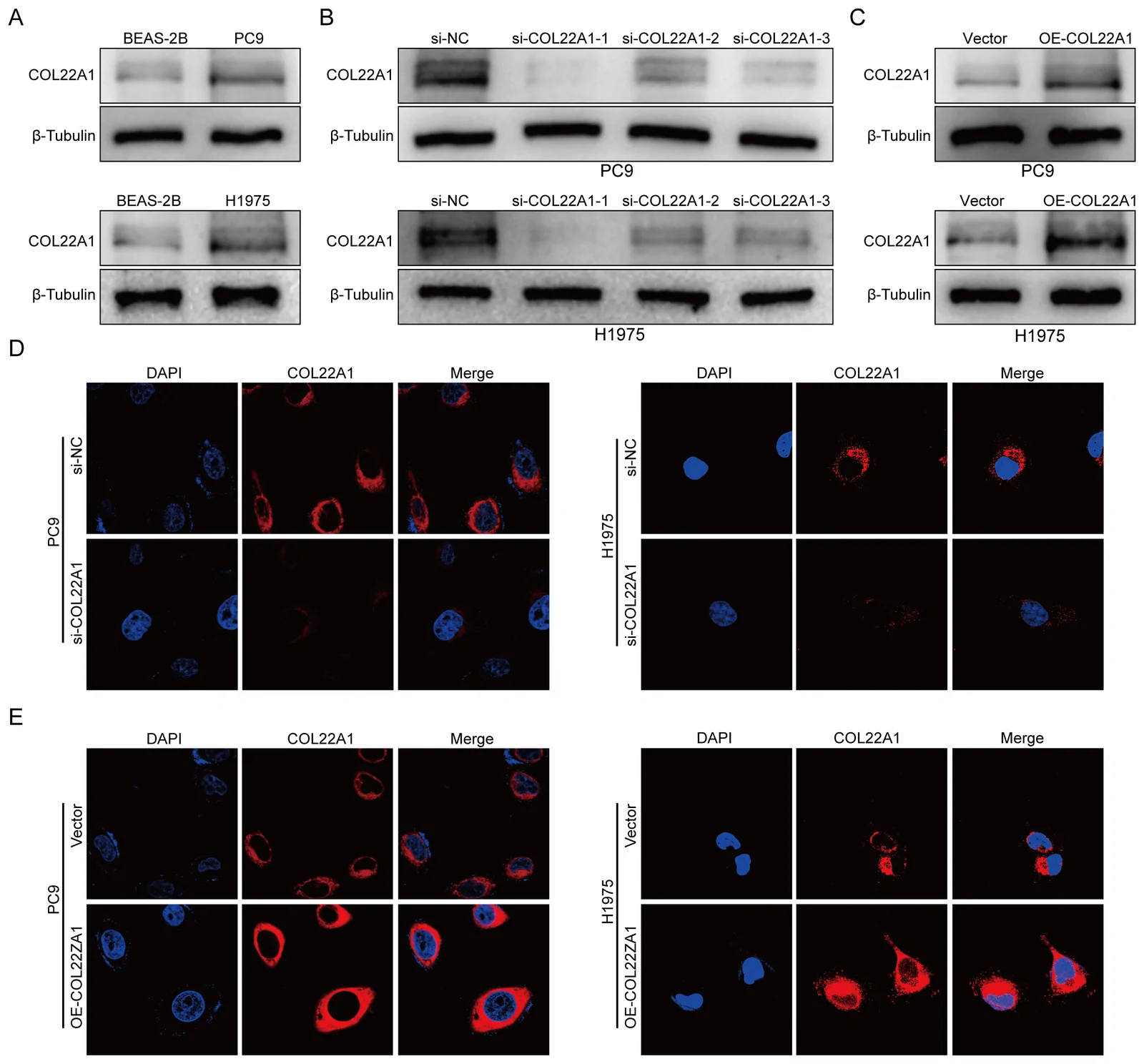
*COL22A1* expression and transfection efficiency in BEAS-2B, PC9, and H1975 cells. **(A)** Basal COL22A1 protein expression detected by Western blot. **(B)** Knockdown efficiency of COL22A1 assessed by Western blot. **(C)** Overexpression efficiency of COL22A1 assessed by Western blotting. **(D)** Immunofluorescence detection of COL22A1 after knockdown. **(E)** Immunofluorescence detection of COL22A1 after overexpression.

CCK-8 results indicated that *COL22A1* depletion inhibited the proliferation of PC9 and H1975 cells, while its overexpression produced the opposite effect (**Figure 8A**). In H1975 cells, colony formation assays showed that *COL22A1* knockdown reduced the number of colonies, whereas *COL22A1* overexpression increased colony formation (**Figure 8B**). Consistently, *COL22A1* depletion lowered EdU positivity, while *COL22A1* overexpression produced the opposite effect (**Figure 8C**; **Supplementary Figure 5A**). In addition, apoptosis assays showed that *COL22A1* knockdown increased the apoptotic rate of PC9 and H1975 cells, whereas *COL22A1* overexpression reduced the apoptotic rate (**Figure 8D**; **Supplementary Figure 5B**). Transwell assays showed that fewer PC9 and H1975 cells migrated or invaded through the membrane after *COL22A1* knockdown (**Figure 8E**; **Supplementary Figure 5C**).

**Figure 8:**
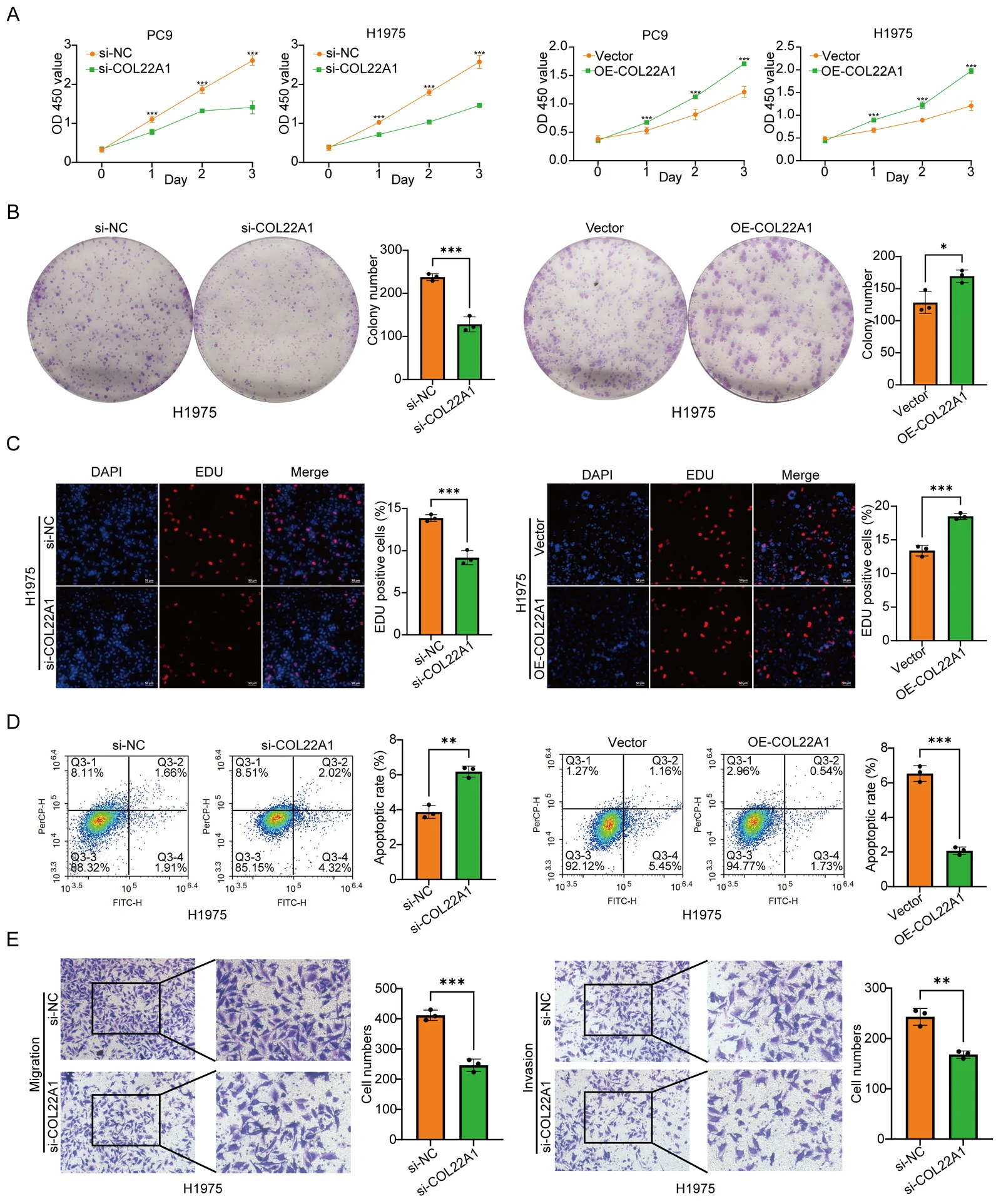
Effects of aberrant *COL22A1* expression on malignant phenotypes of LUAD cells. **(A)** CCK-8 analysis of PC9 and H1975 cell proliferation after *COL22A1* knockdown or overexpression. **(B)** Clonogenic growth of H1975 cells following *COL22A1* knockdown or overexpression. **(C)** EdU staining analysis of H1975 cell proliferation following *COL22A1* knockdown or overexpression. **(D)** Flow cytometric analysis of apoptosis in H1975 cells following *COL22A1* knockdown or overexpression. **(E)** Transwell assays were used to evaluate the migration and invasion abilities of H1975 cells after *COL22A1* knockdown. *p < 0.05; **p < 0.01; ***p < 0.001.

### 3.10 *COL22A1* is involved in FAK/Src signaling regulation

To further explore the potential molecular mechanism by which *COL22A1* participates in LUAD progression, single-gene GSEA was performed for *COL22A1*. GSEA revealed significant enrichment of *COL22A1*-associated genes in the integrin-mediated signaling pathway and focal adhesion assembly, suggesting that *COL22A1* may be associated with focal adhesion-related signal transduction (**Figure 9A**). Given that FAK and Src are key molecules in integrin/focal adhesion signaling, the phosphorylation levels of FAK and Src were further examined.

**Figure 9:**
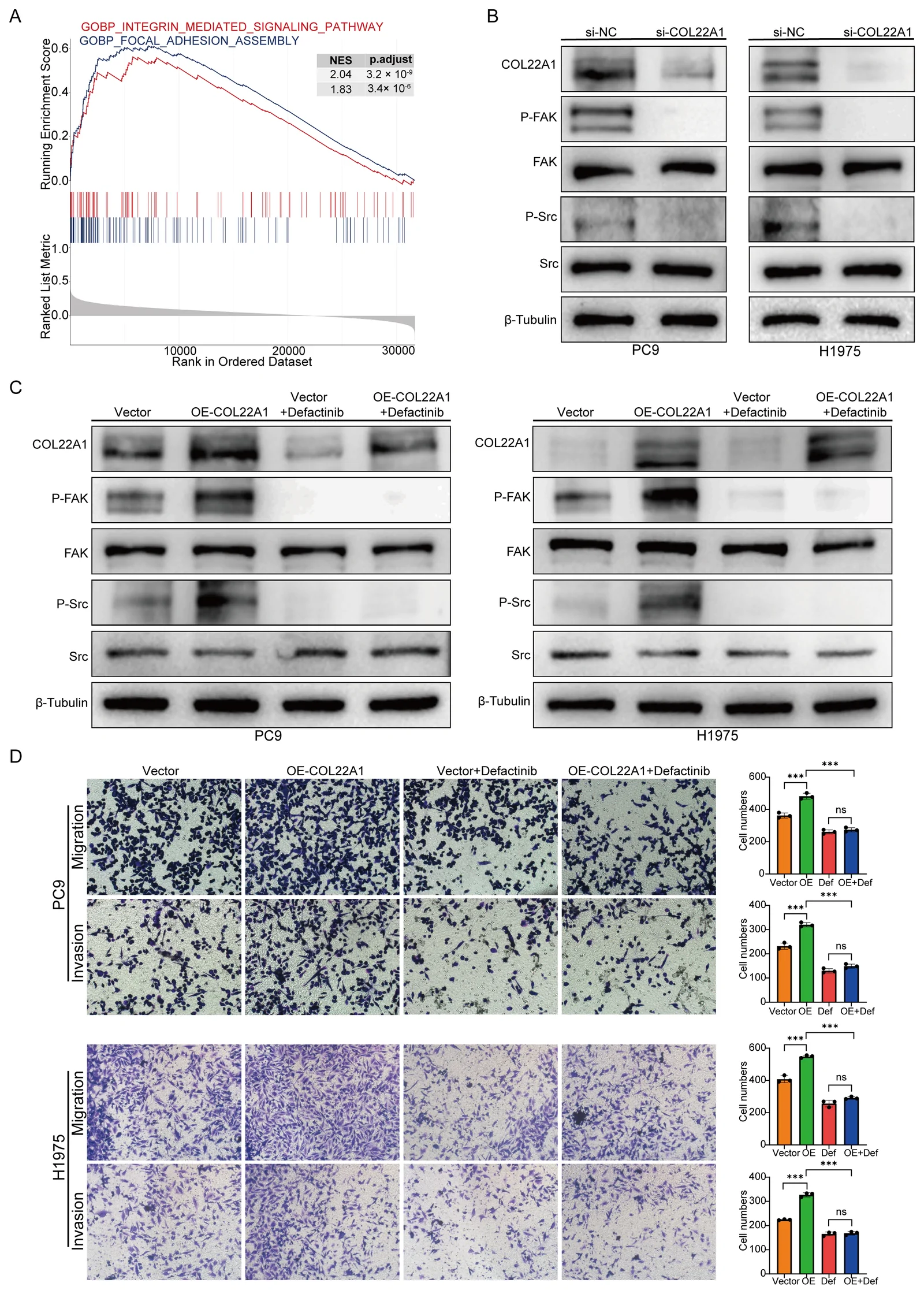
*COL22A1* is involved in FAK/Src signaling regulation. **(A)** Single-gene GSEA of *COL22A1*. **(B)** Western blot analysis of FAK/Src signaling-related proteins in PC9 and H1975 cells after *COL22A1* knockdown. **(C)** Western blot analysis of FAK/Src signaling-related proteins in PC9 and H1975 cells after Defactinib treatment in *COL22A1*-overexpressing cells. **(D)** Transwell assays evaluating the effects of *COL22A1* overexpression on PC9 and H1975 cell migration and invasion following Defactinib treatment. ns, not significant; ***p < 0.001.

Western blot analysis showed that *COL22A1* knockdown markedly decreased p-FAK and p-Src levels in PC9 and H1975 cells (**Figure 9B**). Subsequently, *COL22A1*-overexpressing cells were treated with the FAK inhibitor Defactinib. Western blot analysis revealed that *COL22A1* overexpression enhanced FAK and Src phosphorylation, whereas Defactinib markedly suppressed this effect. Following Defactinib treatment, p-FAK and p-Src remained at similarly low levels in the Vector and OE-*COL22A1* groups (**Figure 9C**). Consistently, Transwell assays demonstrated that *COL22A1* overexpression increased the migratory and invasive capacities of PC9 and H1975 cells, while Defactinib largely abolished these effects. No significant differences were observed between the OE-*COL22A1* + Defactinib and Vector + Defactinib groups (**Figure 9D**).

## 4 Discussion

As a common malignant tumor worldwide, lung cancer continues to impose a substantial public health burden (30). Among its histological subtypes, lung adenocarcinoma (LUAD) is the most prevalent, and improving the prognosis of patients with LUAD remains a major focus of current research. Mechanical stimulation is increasingly regarded as an active driver of malignant progression and tumor immune microenvironment remodeling, rather than merely a byproduct of tumor growth (31–33). We constructed a machine learning-based prognostic model and explored the immune microenvironment characteristics associated with mechanical stimulation-related alterations. We also investigated the function and potential mechanism of the key gene *COL22A1*, providing new insights into the relationship among mechanical stimulation, immune microenvironment remodeling, and LUAD progression.

Using the AUCell algorithm to quantify mechanical stimulation levels, we observed marked cellular heterogeneity in LUAD, with endothelial cells, fibroblasts, and epithelial cells exhibiting relatively high MS scores. In the high-MS group, predicted intercellular communication was increased, especially between fibroblasts and epithelial cells, suggesting that mechanical stimulation may be associated with stromal–epithelial crosstalk within the tumor microenvironment. NicheNet analysis further supported this possibility by indicating that fibroblast-derived signals may influence epithelial cells through pathways related to ECM remodeling, focal adhesion, and PI3K/Akt signaling. These findings provide a possible link between mechanical stimulation and tumor microenvironment remodeling.

Building on these observations, we integrated differential expression profiling, WGCNA, and univariate Cox analysis to screen prognostic MSRGs and subsequently generated a machine-learning-based prognostic model. Across the TCGA cohort and three external GEO cohorts, the model showed stable predictive ability. Zeng et al. developed an ECM organization-related prognostic signature with a 1-year AUC of 0.72, whereas Dong et al. constructed a collagen-based risk model with AUC values ranging from 0.60 to 0.74 across different cohorts (34, 35). In our three external GEO validation cohorts, the AUC values for 1-, 3-, and 5-year OS ranged from 0.63 to 0.76, with the highest AUC exceeding those reported for the aforementioned ECM-related models, suggesting improved predictive performance at certain time points. Moreover, previous ECM- or collagen-related prognostic signatures were mainly developed using bulk transcriptomic data, whereas our model further integrated single-cell-defined mechanical-stimulation states with ssGSEA, WGCNA, and machine-learning analysis. Importantly, immune microenvironment analysis showed that high-risk tumors had reduced T- and B-cell infiltration, higher tumor purity, elevated *CD276* expression, and increased TIDE scores. Collectively, this immune profile is consistent with immune-cold and immune-evasive features in the high-risk group. Although the immune score was significantly lower in the high-risk group, the stromal score did not differ significantly between the two risk groups, indicating that the immune-cold phenotype cannot be explained simply by differences in overall stromal abundance. Because ESTIMATE does not directly capture ECM architecture or mechanical properties, structural ECM remodeling, such as altered collagen organization, may still restrict T-cell trafficking and contribute to immune exclusion. Thus, ECM remodeling may provide a potential mechanistic link between the mechanical stimulation-related risk signature and the immune-cold phenotype of high-risk tumors.

Beyond differences in immune-cell abundance, ECM remodeling and the resulting changes in tissue mechanics may be important determinants of immune exclusion in LUAD. Increased collagen deposition, organization, and crosslinking can increase matrix density and stiffness, thereby restricting T-cell migration and penetration into tumor regions (36, 37). Dense collagen may also suppress T-cell proliferation and cytotoxic activity, while collagen–LAIR1 signaling can promote CD8^+^ T-cell exhaustion and resistance to PD-1/PD-L1 blockade (36, 38); conversely, reducing collagen crosslinking and tumor stiffness has been shown to improve T-cell migration and enhance anti-PD-1 efficacy (37). In support of this ECM–immune relationship, recent multi-omic profiling of NSCLC showed that tumor cells and *SPP1*-positive macrophages interact with *COL11A1*-positive cancer-associated fibroblasts, promoting collagen accumulation at tumor boundaries and restricting T-cell infiltration (39). Beyond ECM mechanics, stromal TGF-β signaling, cytokine-mediated recruitment of immunosuppressive macrophages, and persistent JAK/STAT activation may further reinforce T-cell exclusion or dysfunction and reduce responsiveness to PD-1 blockade (40–42).

This study further identified and validated *COL22A1* as a key mechanical stimulation-related gene in LUAD. In glioma, *COL22A1* overexpression has been implicated in the preservation of malignant phenotypes, partly through activation of PI3K/AKT signaling (43). In this study, *COL22A1* was significantly upregulated in LUAD tissues and was closely associated with clinical stage and poor prognosis. Spatial transcriptomics showed enrichment of *COL22A1* expression in epithelial-rich tumor regions, although contributions from adjacent stromal cells could not be excluded. Single-cell RNA-seq detected *COL22A1* transcripts in epithelial cells, with expression also observed in macrophages, fibroblasts, and monocytes. Functional experiments showed that knockdown of *COL22A1* inhibited proliferation, migration, and invasion and promoted apoptosis in PC9 and H1975 cells, whereas overexpression of *COL22A1* enhanced cell proliferation and reduced apoptosis. In addition, colony formation assays in H1975 cells showed that *COL22A1* knockdown reduced colony formation, while its overexpression produced the opposite effect. These findings suggest that *COL22A1* plays a pro-tumorigenic role in LUAD progression. Single-gene GSEA of *COL22A1* showed that *COL22A1*-related genes were significantly enriched in the integrin-mediated signaling pathway and focal adhesion assembly. Based on the enrichment of integrin-mediated signaling and focal adhesion assembly, we further examined whether COL22A1 regulates FAK/Src signaling. Further experiments showed that *COL22A1* knockdown markedly reduced FAK and Src phosphorylation, whereas *COL22A1* overexpression increased p-FAK and p-Src levels. Following treatment with the FAK inhibitor Defactinib, this signaling activation was markedly attenuated. Functional experiments further showed that Defactinib attenuated the *COL22A1* overexpression-induced enhancement of LUAD cell migration and invasion. Notably, after Defactinib treatment, p-FAK and p-Src levels were similarly low in the Vector and OE-*COL22A1* groups, with no significant differences in migratory or invasive capacity between the two groups. These results suggest that *COL22A1* may promote LUAD progression by modulating the FAK/Src signaling axis.

*COL22A1* encodes the collagen α1(XXII) chain, which assembles into homotrimeric collagen XXII, a member of the FACIT collagen family (44). In non-tumor tissues, collagen XXII is localized at force-transmitting interfaces, and *COL22A1* expression can be induced by mechanical loading, linking it to mechanical force transmission and responsiveness (45–47). In glioblastoma, *COL22A1* overexpression has also been associated with MMP-2/MMP-9 upregulation and ECM remodeling, suggesting a potential role in modifying the mechanical tumor microenvironment (48). Collagen XXII can interact with the collagen-binding integrins α1β1, α2β1, α10β1, and α11β1, with direct binding particularly demonstrated for α2β1 (49). Considering that α2β1 has also been implicated in collagen-dependent invasion in A549 lung adenocarcinoma cells, we speculate that it may represent a putative receptor for COL22A1 in LUAD cells (50). Our enrichment analysis further identified integrin-mediated signaling and focal adhesion assembly as prominent *COL22A1*-associated processes. Integrins function as mechanoreceptors that connect the ECM to the actin cytoskeleton and transmit extracellular mechanical cues through focal adhesions, with the FAK/Src axis serving as a major downstream mechanosensitive pathway (51, 52). Consistent with this mechanism, our experiments showed that *COL22A1* manipulation altered FAK and Src phosphorylation. Collectively, these findings indicate that COL22A1 may influence ECM–integrin coupling and participate in FAK/Src-associated mechanotransduction, thereby promoting malignant cellular behaviors in LUAD.

This study combined multi-omics analyses with experimental validation, but several limitations remain. Our conclusions were largely based on public datasets and *in vitro* experiments and should therefore be further validated in animal models and larger clinical cohorts. Moreover, the molecular details by which COL22A1 regulates FAK/Src signaling remain unclear. Although our integrative analyses and functional findings support a potential role for COL22A1 in mechanotransduction, future studies should directly examine whether mechanical stimulation regulates *COL22A1* expression and whether collagen XXII alters matrix stiffness in LUAD. In addition, the immune-related findings were mainly derived from computational analyses and require further validation in clinical immunotherapy cohorts and experimental models.

Our findings delineate the expression features, cell–cell communication patterns, and immune microenvironment characteristics associated with mechanical stimulation-related genes in LUAD. A reliable prognostic model was constructed, and high-risk tumors exhibited immune-cold and immune-evasive features. *COL22A1* was identified as a potential driver of LUAD progression through FAK/Src signaling, providing complementary evidence for patient stratification, immune microenvironment assessment, and future therapeutic investigation.

## Data Availability

Publicly available datasets were analyzed in this study. This data can be found here: GSE72094, GSE31210, GSE50081, GSE189357, GSM9226190, https://codeocean.com/capsule/8321305/tree/v1.
