# Supplementary material for: A mechanical stimulation-related gene signature predicts prognosis, reflects immune microenvironment remodeling, and identifies COL22A1 as a potential therapeutic target in lung adenocarcinoma
Source: Front Immunol. 2026 Sep 16;17:1918878. doi: 10.3389/fimmu.2026.1918878 (PMC13624094; doi:10.3389/fimmu.2026.1918878)

Figure 7A

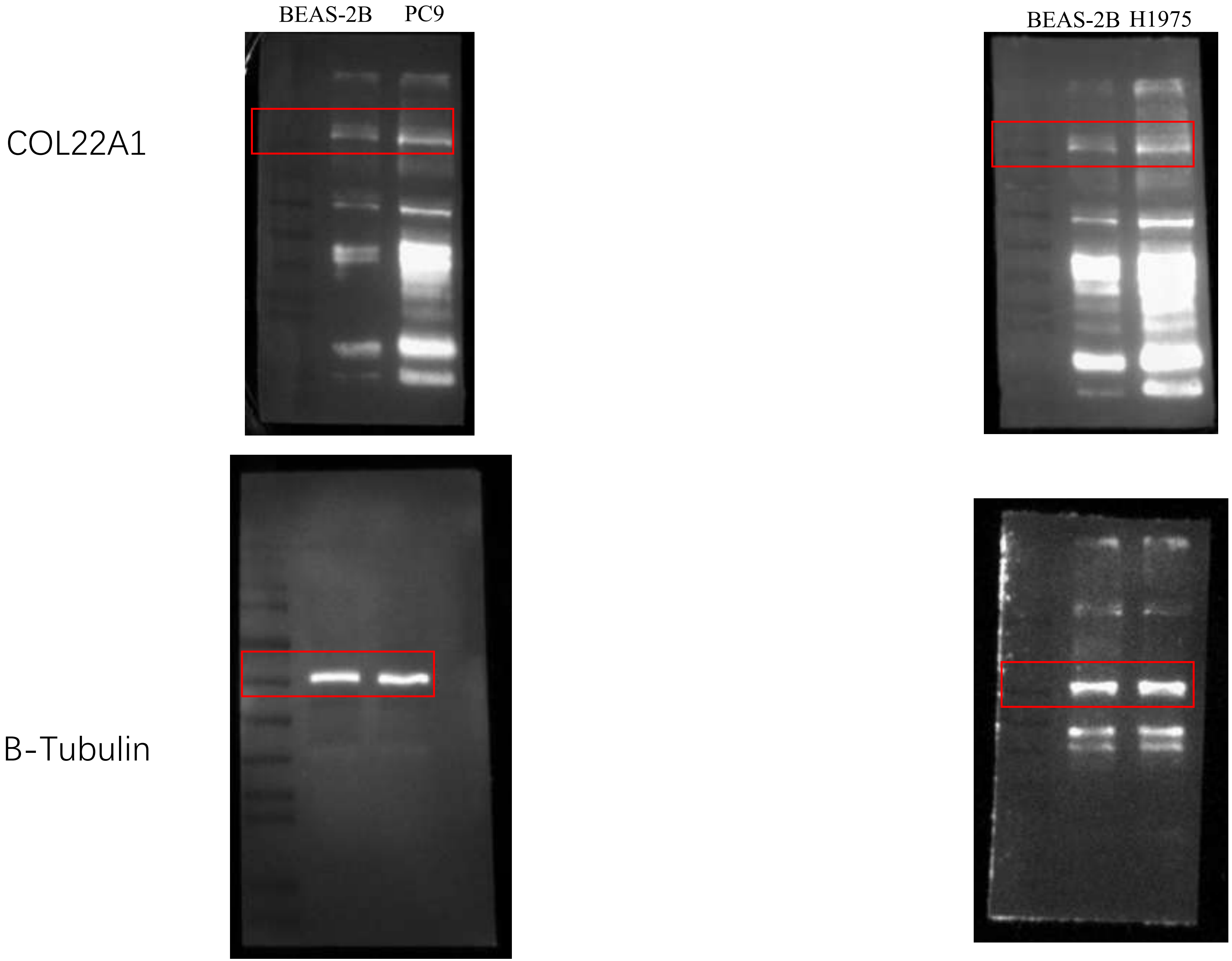

Figure 7B

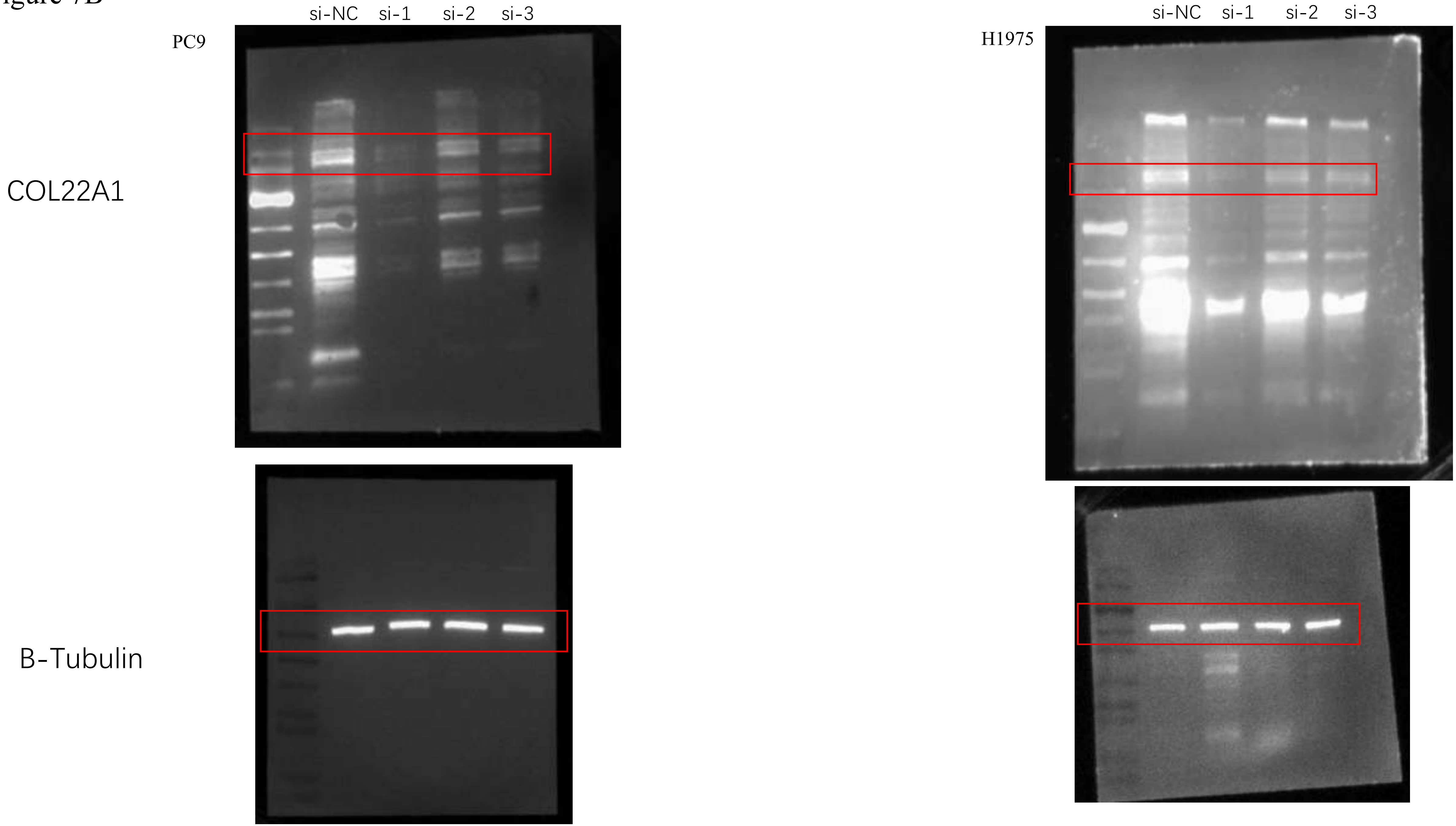

Figure 7C

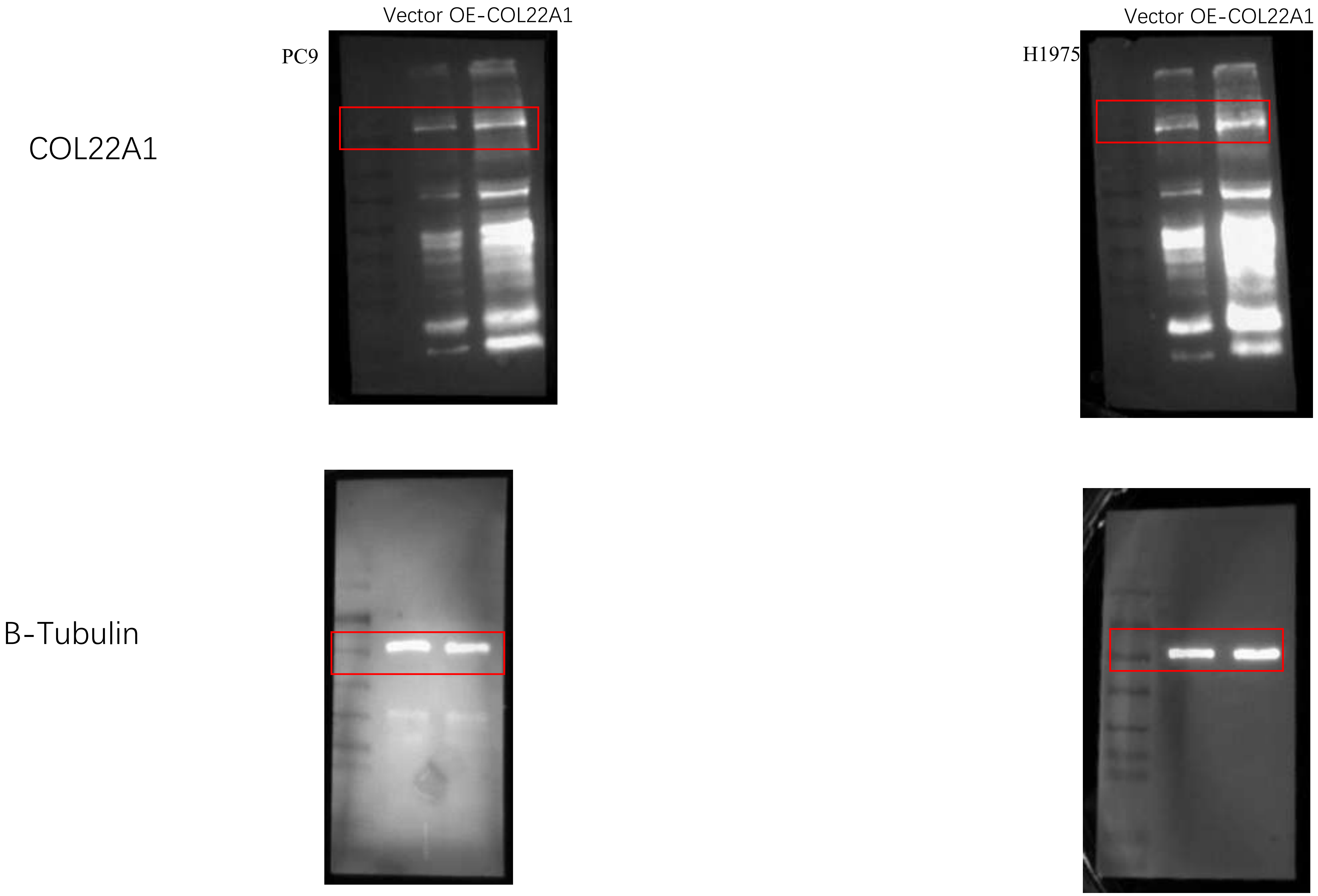

Vector OE-COL22A1
PC9
COL22A1
B-Tubulin
Vector OE-COL22A1
H1975

Figure 9B

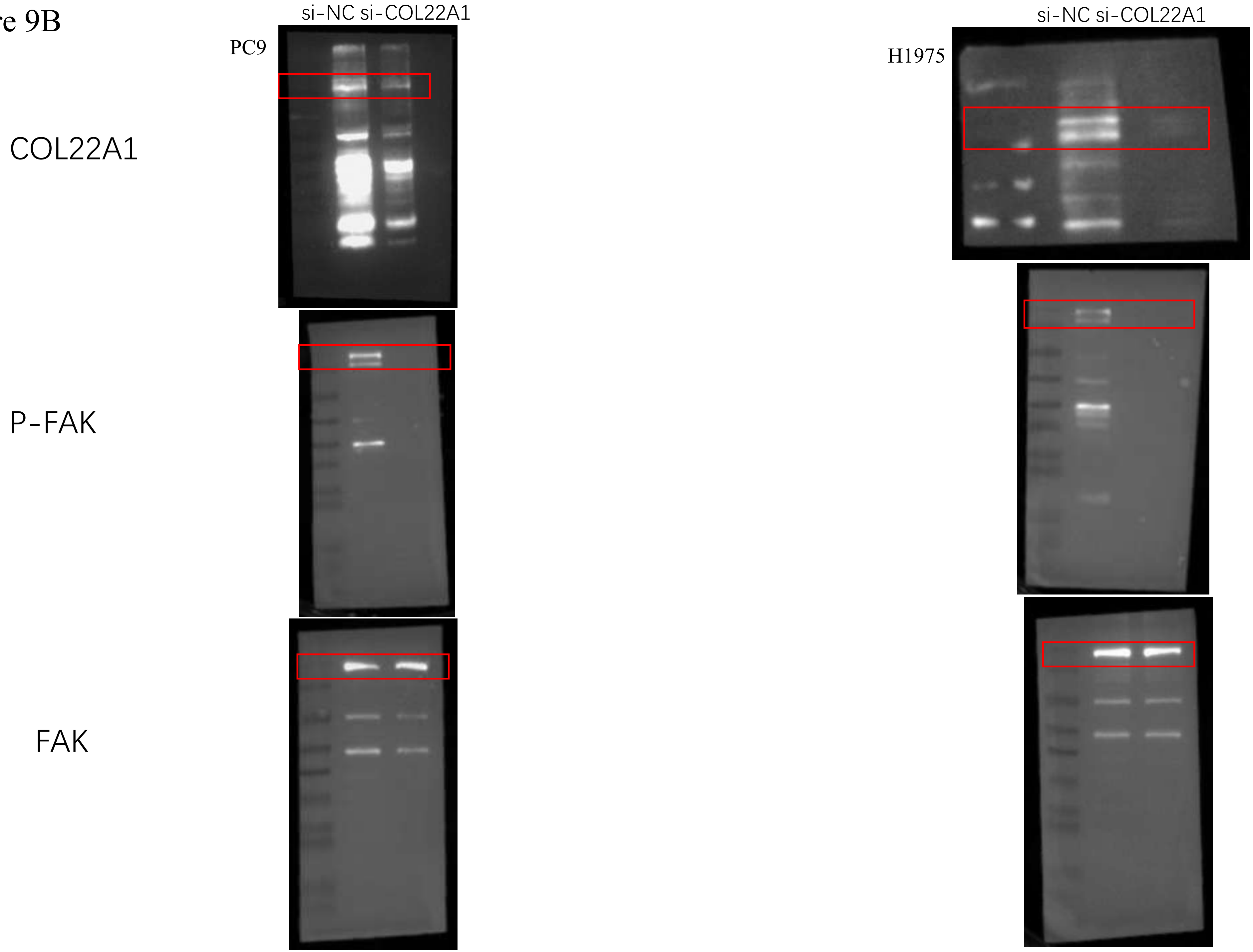

Figure 9B

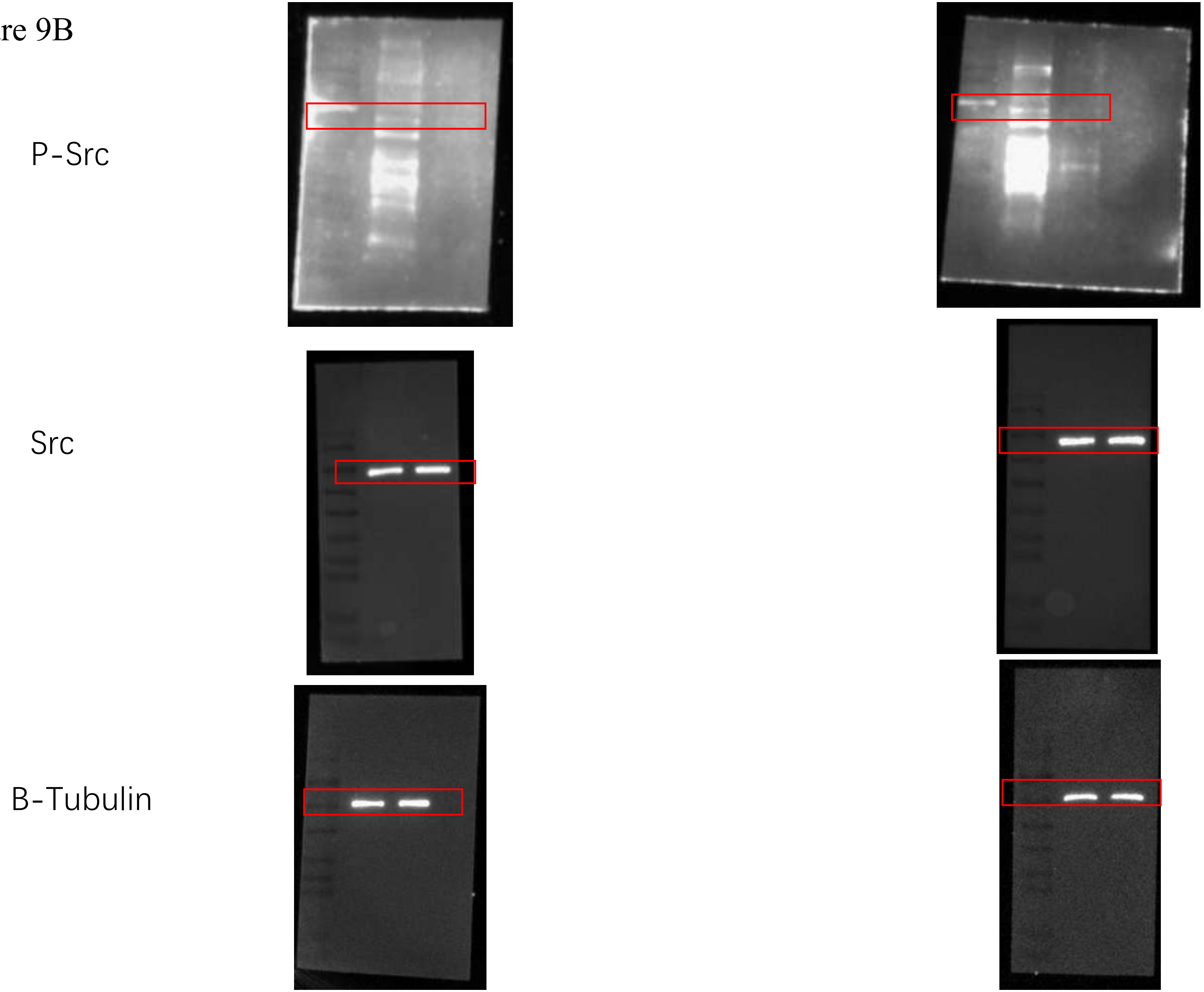

Figure 9C

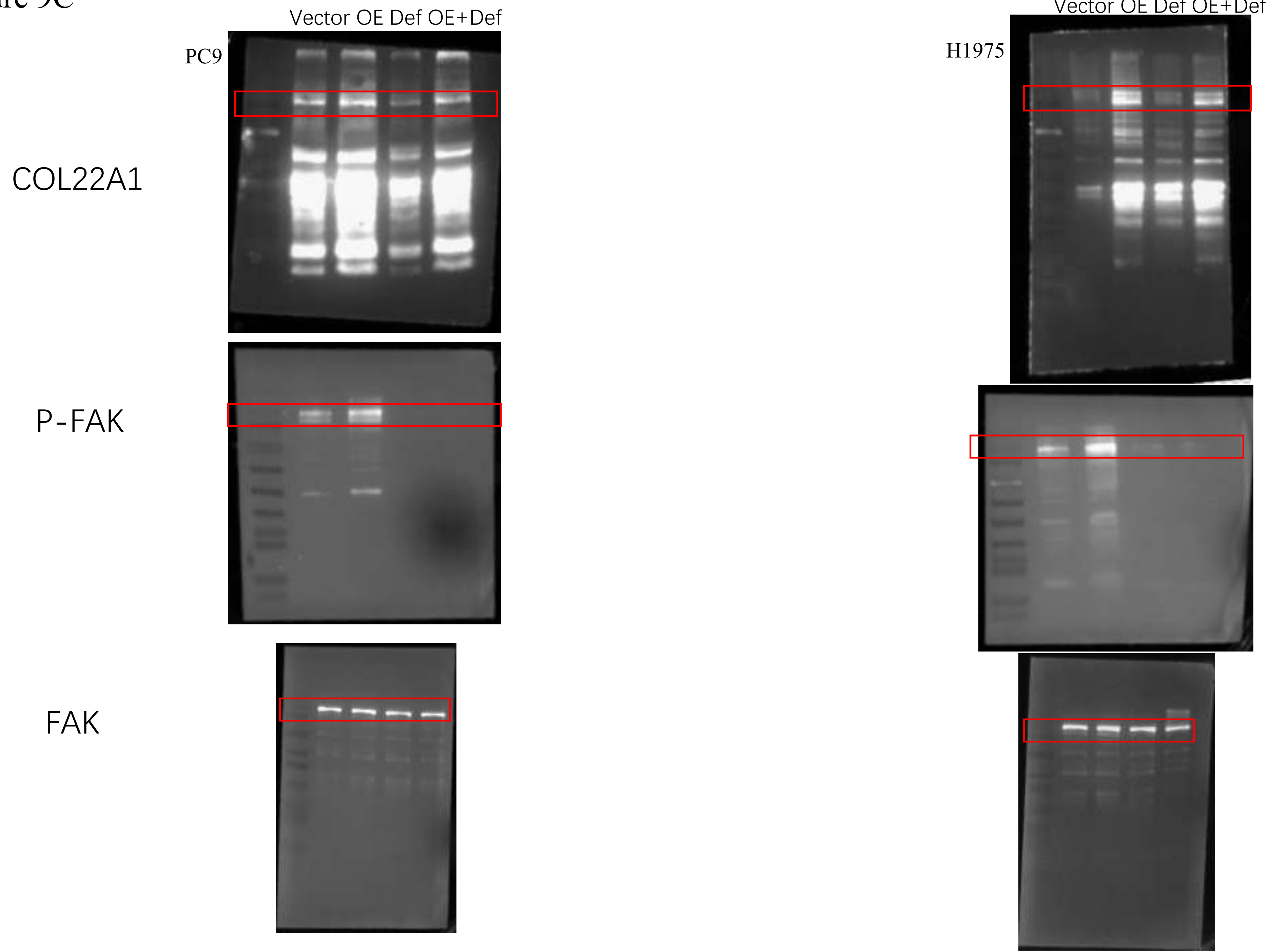

PC9
Vector OE Def OE+Def
COL22A1
P-FAK
FAK
H1975
Vector OE Def OE+Def

Figure 9C

P-Src

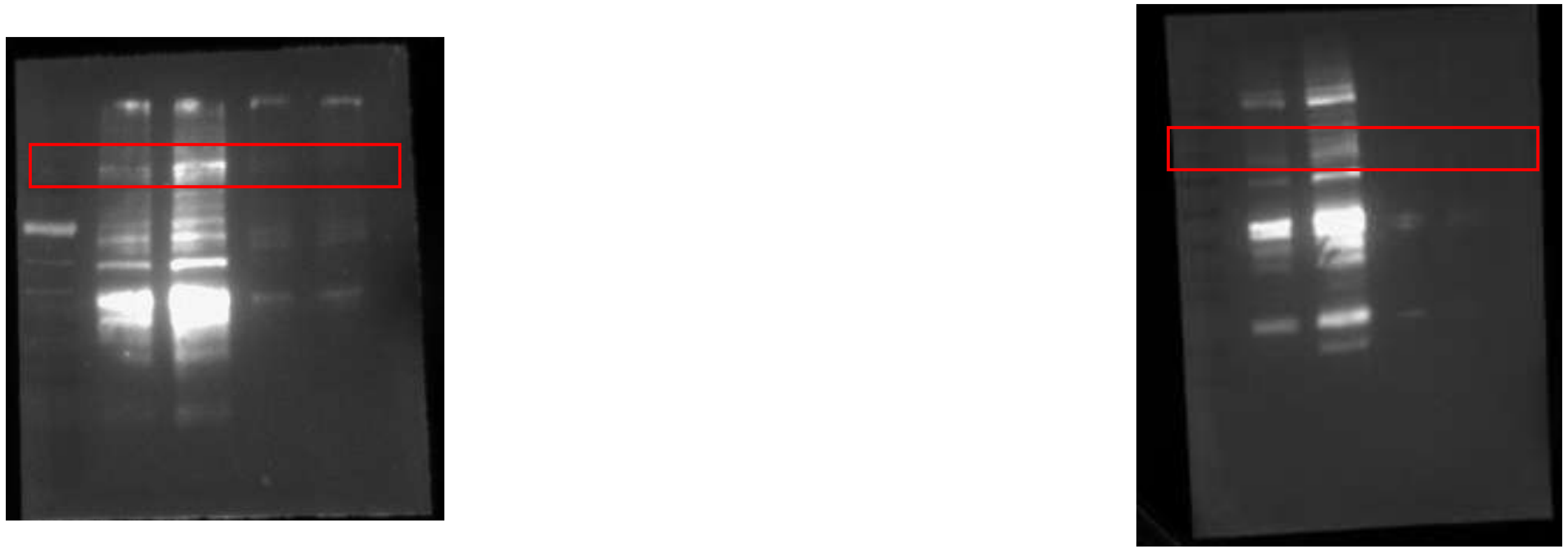

Src

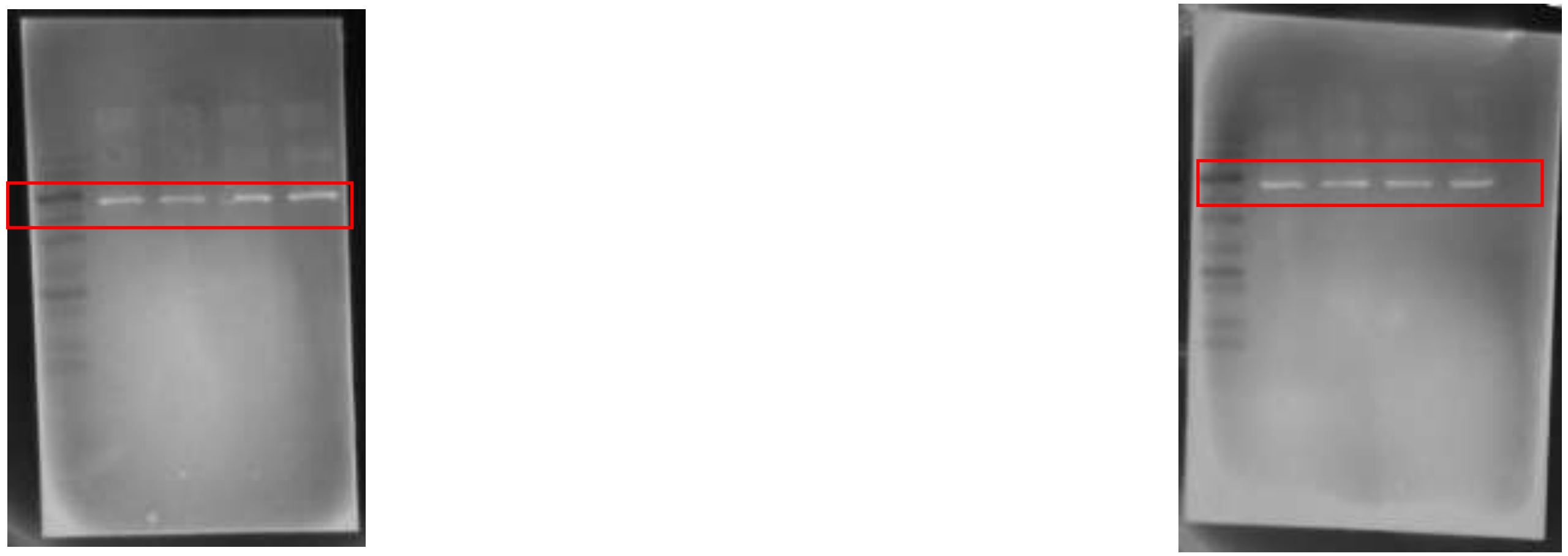

B-Tubulin

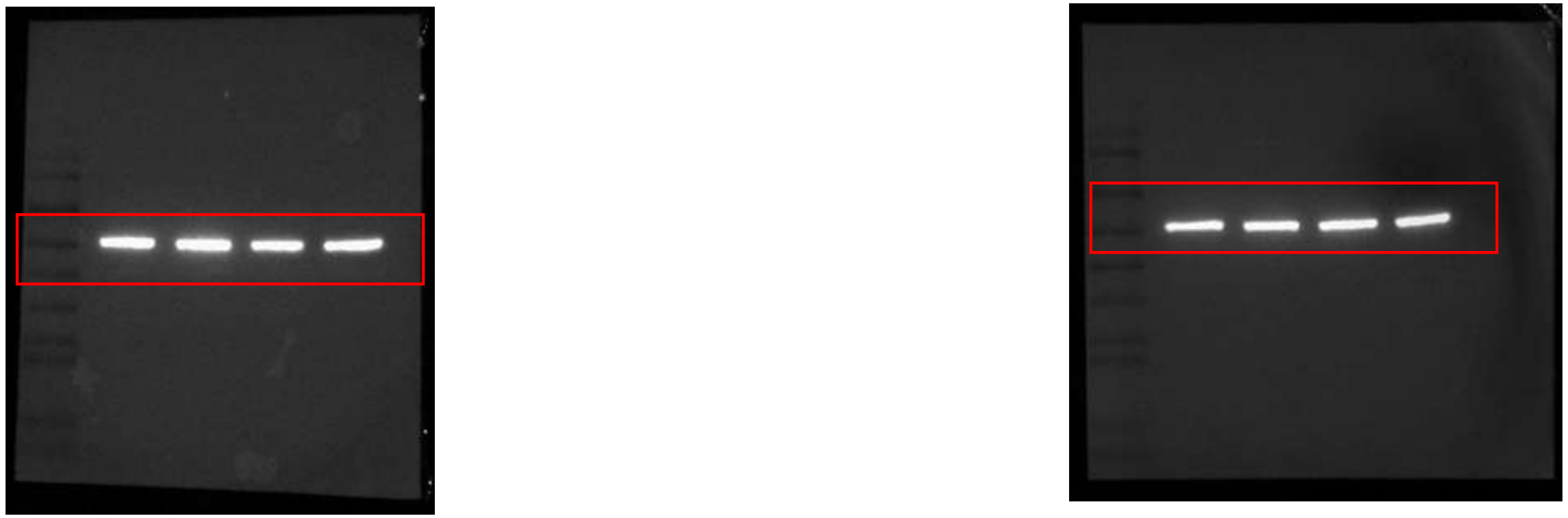

Supplement: Supplementary file 1 [file DataSheet1.pdf]
