# Supplementary material for: A mechanical stimulation-related gene signature predicts prognosis, reflects immune microenvironment remodeling, and identifies COL22A1 as a potential therapeutic target in lung adenocarcinoma
Source: Front Immunol. 2026 Sep 16;17:1918878. doi: 10.3389/fimmu.2026.1918878 (PMC13624094; doi:10.3389/fimmu.2026.1918878)
Supplement: Supplementary file 2 [file Table1.docx]

**Supplementary Table**

**Supplementary Table 1*.*** **siRNAs sequences used in COL22A1 knockdown experiment.**

| Name | Sense or antisense | siRNA sequence |
| --- | --- | --- |
| COL22A1 siRNA-1 | sense sequence | 5ʹ-  UGAUGGUCUUCAAAAACCCAA -3ʹ |
|  | antisense sequence | 5ʹ-  GGGUUUUUGAAGACCAUCAAC -3ʹ |
| COL22A1 siRNA-2 | sense sequence | 5ʹ-  AAAACCUGUGAUUUCCUUGGU -3ʹ |
|  | antisense sequence | 5ʹ-  CAAGGAAAUCACAGGUUUUGA -3ʹ |
| COL22A1 siRNA-3 | sense sequence | 5ʹ- UUUUCCCUUCUGUGCCUUCUC-3ʹ |
|  | antisense sequence | 5ʹ- GAAGGCACAGAAGGGAAAAAG-3ʹ |
