# Supplementary material for: A mechanical stimulation-related gene signature predicts prognosis, reflects immune microenvironment remodeling, and identifies COL22A1 as a potential therapeutic target in lung adenocarcinoma
Source: Front Immunol. 2026 Sep 16;17:1918878. doi: 10.3389/fimmu.2026.1918878 (PMC13624094; doi:10.3389/fimmu.2026.1918878)
Supplement: Supplementary file 3 [file Table2.docx]

**Supplementary Figures**

*Supplementary Figure S1.* **Mechanical stimulation-related genes in single-cell data. (A) Overlap between MSRGs and marker genes from the single-cell dataset. (B) AUCell scores of mechanical stimulation across different cell types.**

*Supplementary Figure S2*. Communication between fibroblasts and epithelial cells. (A) Ranking of predicted ligand activity derived from fi-broblasts. (B) Heatmap of predicted ligand-target regulatory relationships. (C) KEGG pathway enrichment of the predicted target genes. (D) GO enrichment of the predicted target genes.

*Supplementary Figure S3*. Selection of the LASSO + RSF model. (A) LASSO coefficient profiles across different values of log(λ). (B) Error rate of the Random Survival Forest (RSF) model according to tree number.

*Supplementary Figure S4*. Nomogram-based survival prediction in LUAD. (A) Univariate Cox analysis of clinicopathological variables and risk score. (B) Multivariate Cox analysis of clinicopathological variables and risk score. (C) Nomogram integrat-ing risk score and clinical stage. (D) Calibration curves for 1-, 3-, and 5-year survival prediction. (E) Kaplan–Meier curves in the TCGA-LUAD cohort according to nomogram-based risk stratification. (F) Time-dependent ROC curves of the nomogram.

*Supplementary Figure S5*. Effects of aberrant *COL22A1* expression on proliferation, apoptosis, migration, and invasion in PC9 cells. (A) EdU staining analysis of PC9 cell proliferation following *COL22A1* knockdown or overexpression. (B) Flow cytometric analysis of apoptosis in PC9 cells after *COL22A1* knockdown or overexpression. (C) Transwell assays were used to evaluate the migration and invasion abilities of PC9 cells following *COL22A1* knockdown. ***p* < 0.01; ****p* < 0.001.
